# Comparative Transcriptome Analysis of Two Chrysomycin-Producing Wild-Type and Mutant Strains of *Streptomyces* sp. 891

**DOI:** 10.3390/metabo12121170

**Published:** 2022-11-24

**Authors:** Wangjie Zhu, Xinwei Pei, Xiaoyu Chen, You Wu, Fuhang Song, Huawei Zhang

**Affiliations:** 1 School of Pharmaceutical Sciences, Zhejiang University of Technology, Hangzhou 310014, China; 2 Department of Light Industry, Beijing Technology and Business University, Beijing 100048, China

**Keywords:** *Streptomyces*, chrysomycin, comparative transcriptome analysis, mutant strain, differentially expressed genes (DEGs)

## Abstract

Chrysomycin A (CA), a promising antibiotic agent, usually coexists with two analog chrysomycins B (CB) and C (CC) produced by several wild-type (WT) *Streptomyces* strains. With the aim to increase CA production, UV mutagenesis-based breeding had been employed on a marine-derived strain *Streptomyces* sp. 891 in our previous study and afforded an improved strain 891-B6 with enhanced CA yield. By comparative transcriptome analysis, significant differences in chrysomycin BGC-related gene expression between the WT strain 891 and the mutant strain 891-B6 were unveiled in the current study. Among 25 up-regulated genes in mutant 891-B6, *chryA*, *chryB*, *chryC*, *chryF*, *chryG*, *chryK*, *chryP*, and *chryQ*, responsible for the biosynthesis of benzonaphthopyranone aglycone, and *chryD, chryE*, and *chryU* in charge of production of its deoxyglycoside, were characterized. Furthermore, the expression of genes *chryOII*, *chryOIII*, and *chryOIV* responsible for the formation of 8-vinyl in CA from 8-ethyl in CB were greatly enhanced in strain 891-B6. These findings provide molecular mechanisms for increased yield of CA and decreased yield of CB for mutant 891-B6, which has potential application in industrial CA production.

## 1. Introduction

**Figure 1:**
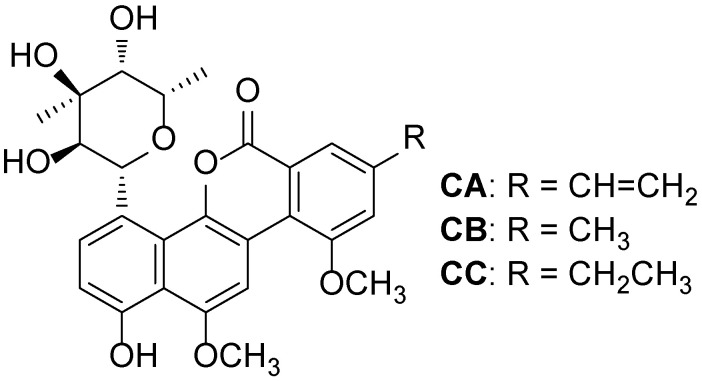
Chemical structures of chrysomycins A-C (CA-CC).

Chrysomycins A-C (CA-CC, Figure 1), one group of glycosides consisting of a special heptose and a benzonaphthopyranone aglycone, were originally discovered from strain *Streptomyces* sp. A-419 in 1955 by Strelitz and coworkers [1]. Up to now, these substances had been isolated and identified from other *Streptomyces* strains, including 891 [2], *S. sporoverrucosus* MTCC11715 [3], *S. albaduncus* C38291 [4], *S. arenae* [5], and OA161 [6]. A number of pharmacological studies showed that chrysomycin A (CA) displayed excellent antibacterial activity toward multi-drug-resistant (MDR) and extreme-drug-resistant (XDR) tuberculosis (TB), methicillin-resistant *Staphylococcus aureus* (MRSA), *B. cereus* and *Bacillus subtilis* [1,5,6,7,8] as well as strong cytotoxicity against human lymphocytic leukemia HL-60 cells, glioblastoma U251, and U87-MG cells [3,9,10,11]. Moreover, it exhibited excellent anti-neuroinflammatory effects by down-regulating NLRP3/Cleaved Caspase-1 signaling pathway [12]. Therefore, CA is an important and promising drug candidate for the treatment of Gram-positive bacterial infections and anti-tumor therapies.

Strain 891 was originally isolated from marine sediments and found to produce CA, CB, and CC with a total yield of 1.7 g/L in the ratio of 71:22:7 (Figure S1a). Their biosynthetic gene cluster (BGC) was characterized by whole-genome sequencing and antiSMASH analysis, and their biosynthesis initiate condensation of two activated substrates (propionyl-CoA and acetyl-CoA) and nine malonyl-CoAs as extending units followed by a series of successive oxidation, reduction, rearrangement [13]. In order to improve CA yield for new drug development, UV mutagenesis was employed to improve strain 891 and afforded a mutant strain 891-B6 with the CA:CB:CC ratio of 83:8:9 (Figure S1b). RNA sequencing (RNA-Seq)-based comparative transcriptome analysis has emerged as an essential method for the identification of differentially expressed genes (DEGs) in mutant strains, such as DHA-enhanced producing mutant *Aurantiochytrium* sp. X2, lysostaphin-resistant *S. aureus* Newman 1801_2010 and salt-resistant *Saccharomyces cerevisiae* mutant strain [14,15,16,17]. In the current study, this approach was used to unveil the differences in chrysomycin BGC-related gene expression between strains 891 and 891-B6 and to provide insight into the molecular mechanism underlying CA accumulation by a combination of various bioinformatics methods, including DEGs, single nucleotide polymorphisms (SNPs) and insertion-deletion mutations (InDels) analyses.

## 2. Materials and Methods

### 2.1. Microbes and Cultivation

Strain *Streptomyces* sp. 891 was isolated from mangrove sediments in the South China Sea and was frozen in glycerol (GenBank accession No. CP050693-CP050694) at Zhejiang University of Technology (Hangzhou, China). Strain 891-B6 was a mutant of the WT strain 891 through UV mutagenesis as described previously [18], and was stored at China General Microbiological Culture Collection Center (CGMCC No.21775) (Beijing, China). For cultivation, each strain was inoculated onto ISP_2_ agar plate (glucose 4 g/L, yeast extract 4 g/L, malt extract 10 g/L and agar 20 g/L) at 28 °C. Then, a single colony was inoculated in ISP_2_ liquid medium followed by cultivation at 28 °C and 200 rpm for 3 days and RNA extraction. The yield of CA in strains 891 and 891-B6 was determined as 1.2 g/L and 1.7 g/L, respectively.

### 2.2. RNA Extraction

For RNA extraction, the logarithmic growth stage bacterial solutions of strains 891 and 891-B6 were centrifuged at −4 °C and rapidly frozen in liquid nitrogen (−196 °C) for 30 min, and then stored at −80 °C. The gel electrophoretic and HPLC diagrams of RNA samples were shown in Figures S2 and S3. Transcriptome sequencing was performed by Nanjing Personalbio Gene Technology Co., Ltd. (Nanjing, China).

### 2.3. Illumina Sequencing, Assembly, and Annotation

The next-step generation sequencing platform Illumina (Illumina, San Diego, CA, USA) provided the raw data of FASTQ (http://en.wikipedia.org/wiki/FASTQ_format, accessed on 20 February 2022). All RNA-Seq raw data for strains 891 and 891-B6 were analyzed briefly and shown in Table S1. The obtained reads were filtered according to the quality parameters of GC content, sequence duplication level, Q20, and Q30. High-quality clean data for strains 891 and 891-B6 were chosen from raw reads with the average quality score higher than Q20 (Table S2). The single base mass distribution map was used to evaluate the quality of a single nucleobase. The base mass of most sequences was more than 20, indicating that the sequencing quality is high (Figure S4). The reference genome index was established by Bowtie2 (http://bowtie-bio.sourceforge.net/index.shtml, accessed on 22 February 2022), and the filtered Reads were aligned to the reference genome by using Bowtie2. The gene alignment rate of all samples was more than 95%, indicating that the transcriptome sequencing analysis results were reliable (Table S3).

### 2.4. Bioinformatics Analysis

For RNA-seq gene expression data analysis, the HTSeq 0.6.1 p2 (http://www-huber.embl.de/users/anders/HTSeq, accessed on 25 February 2022) tool was used for the statistical comparison of each gene on Read Count value, using fragments per kilobases of transcript per million mapped fragments (FPKM) to map the reads from RNA-seq and the correlation of genes between two samples were assessed by Pearson correlation coefficient. Compared the reads per kilobase per million mapped reads (RPKM) value obtained from the sampling of the sequencing data with all the sequencing data to obtain the relative error, which decreases with the increase of sampling proportion (Figure S5). RNA-seq data were used to compare DEGs, SNPs, and InDels of strains 891 and 891-B6, and to analyze up-regulated and down-regulated genes. The DESeq package of R software (http://www.bioconductor.org/packages/release/bioc/html/DESeq.html, accessed on 26 February 2022) was used to analyze the DEGs and the filter conditions are as follows: |log2FoldChange| > 1, *p*-value < 0.05 [19] (Table S4). Gene Ontology (GO) and Kyoto Encyclopedia of Genes and Genomes (KEGG) pathway enrichment analyses were performed to annotate the potential functions of DEGs between strain 891 and mutant strain 891-B6 [20]. KEGG pathway was mainly analyzed by KEGG’s KAAS (v2.1, https://www.genome.jp/tools/kaas/, accessed on 26 February 2022) automated annotation system and GO pathway was completed by Blast2GO software (v1.0, https://www.blast2go.com/, accessed on 26 February 2022), which the criterion for significant enrichment was *p*-value < 0.05. SNPs and InDels analyses were obtained using Varscan program (v2.3.9, http://varscan.sourceforge.net, accessed on 28 February 2022) with the *p*-value of less than 0.01. SNPs are genetic markers formed by single nucleotide variations in the genome, including transition (A/G, T/C) and transversion (A/T, A/C, G/T, G/C). InDels refer to the insertion or deletion of a small fragment in a sample relative to the reference genome [21].

## 3. Results

### 3.1. Morphological Features of Strains 891 and 891-B6

**Figure 2:**
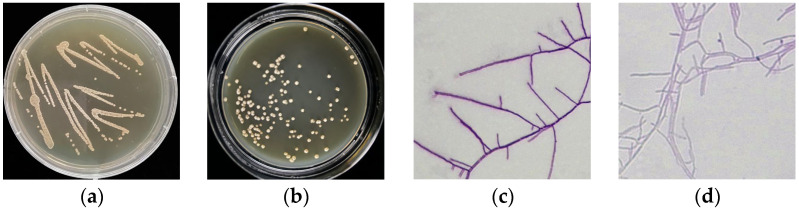
Colonies of strains 891 (**a**) and 891-B6 (**b**) and their mycelia (**c**,**d**).

The 16S rRNA gene-based molecular analysis had displayed that the wild-type (WT) strain 891 belongs to the genus *Streptomyces* [13]. The colonies of strain 891 and mutant 891-B6 grown onto ISP_2_ media were opaque spheres with wrinkles on the surface and depressions in the center of the colonies. Additionally, the mycelia of strain 891-B6 stained by crystal violet had more branches than those of its wild-type strain 891 (Figure 2).

### 3.2. RNA-Seq Gene Expression Analysis

**Figure 3:**
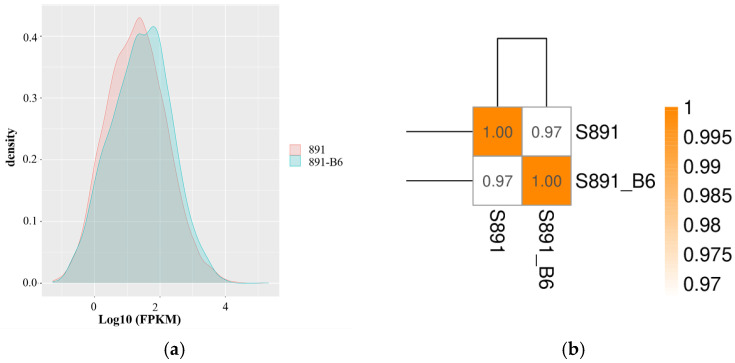
Analysis of the reads from RNA-Seq for strains 891 and 891-B6: (**a**) the map of FPKM density distribution; (**b**) the test results of sample correlation.

As shown in Figure 3a, density distributions of FPKM values created by HTSeq 0.6.1 p2 tool showed that the expression level of most unigenes in strains 891 and 891-B6 was between 0 and 4. The correlation coefficient for these two strains was 0.97, indicating the level of their gene expression was very similar (Figure 3b).

### 3.3. Analyses of DEGs and Identification of Regulated Genes

**Figure 4:**
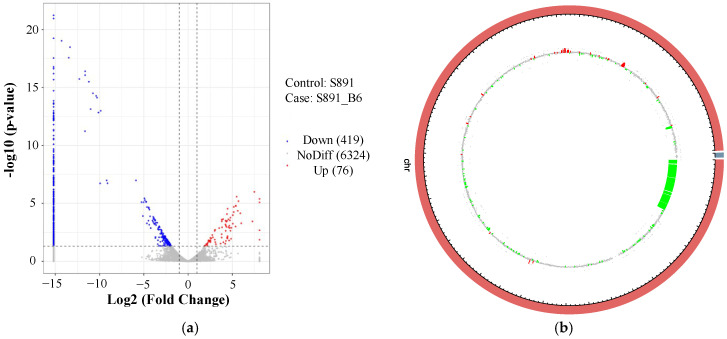
Analysis of DEGs for strain 891-B6: (**a**) the volcano map of the DEGs. Red dots indicate up-regulated genes in this group, blue dots indicate down-regulated genes, and gray dots indicate non-significant genes. (**b**) the genome map of the DEGs. The outer circle is the chromosome band, the red and green bar graphs in the inner circle are up-regulated and down-regulated genes, respectively. The gray dots indicate genes that are not differentially expressed.

A total of 495 DEGs were detected in this study, of which 419 were down-regulated and 76 were up-regulated in strain 891-B6, suggesting its gene transcription level is lower than that of strain 891 (Figure 4). Interestingly, 25 up-regulated genes (Table 1) involved in chrysomycin biosynthesis were discovered in the mutant 891-B6, including *chryA*, *chryB*, *chryC*, *chryF*, *chryG*, *chryK*, *chryP*, *chryQ*, *chryD*, *chryE*, *chryU*, *chryOII*, *chryOIII*, *chryOIV*, and other genes [13,22]. Additionally, the LogFC value (8.07) of *chryB* responsible for encoding chain length factor (KS_β_) is the highest, and that of *chryG* responsible for encoding cyclase to form keratocyclization reaches up to 6.01. Therefore, these up-regulated genes could promote production of a benzonaphthopyranone skeleton of chrysomycin in the mutant 891-B6.

Meanwhile, three up-regulated genes (*chryD, chryE* and *chryU*) used for the accumulation of deoxyglycosides were unveiled in strain 891-B6. Additionally, all putative monooxygenase-encoding genes *chryOII*, *chryOIII*, and *chryOIV* were up-regulated. Particularly, *chryOIII* was determined to be responsible for encoding P450 oxidase, which catalyzes the formation of 8-vinyl in CA from 8-ethyl in CB and enhances CA production [23]. These results showed all up-regulated genes in the mutant 891-B6 are positively related to the biosynthesis of chrysomycins.

### 3.4. GO and KEGG Pathway Enrichment Analyses of DEGs

**Figure 5:**
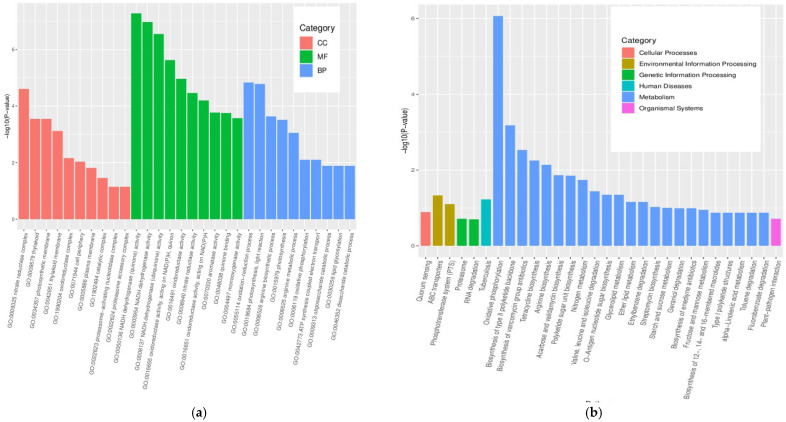
The DEGs of comparative transcriptomic analysis between strains 891 and 891-B6: (**a**) GO; (**b**) KEGG.

**Figure 6:**
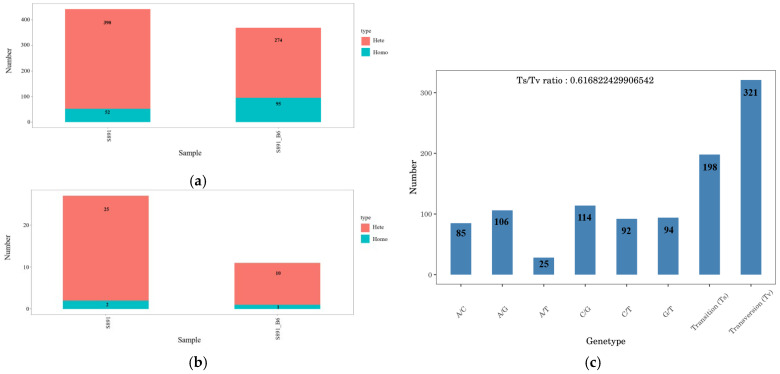
The SNPs (**a**) and InDels (**b**) results for strains 891 and 891-B6; (**c**) transition and transversion results.

To annotate the potential functions of the DEGs in strains 891 and 891-B6, DEGs with >2-fold expression changes were assigned to different enrichment pathways, including GO and KEGG pathway (Figure 5). The GO analysis of the transcriptome consists of three main categories, including biological processes (BP), cellular components (CC), and molecular functions (MF). As shown in Figure 6a, the largest groups in the BP category are oxidation-reduction process, photosynthesis, and light reaction. In the CC category, nitrate reductase complex, thylakoid, and photosynthetic membrane are the dominant groups, while the MF category is mainly enriched in NADH dehydrogenase (quinone) activity [24]. Among these KEGG pathways, metabolism (oxidative phosphorylation and biosynthesis of type II polyketide backbone) has the highest number of genes. In comparison with pathway enrichment analyses of strain 891, the DEGs mainly focused on the oxidation-reduction process and biosynthesis of secondary metabolites. These results suggested that the DEGs in strains 891 and 891-B6 are mainly reflected in the redox reactions in vivo, including the metabolism of substances and the synthesis of secondary metabolites, such as chrysomycins.

### 3.5. Analyses of SNPs and InDels

The SNPs results showed that strain 891 harbors 390 heterozygous variants (Hete) and 52 homozygous variants (Homo), and the mutant 891-B6 has 284 Hete and 95 Homo (Figure 6a). For InDels, there are 25 Hete and 2 Homo in strain 891, while 10 Hete and 1 Homo are in strain 891-B6 (Figure 6b). The number of variants in strain 891 is greater than that of strain 891-B6, indicating that there are more point mutations in strain 891.

In SNPs, there are 198 transitions and 321 transversions in strain 891-B6 (Figure 6c). C/G transversion has the largest number of genes while the number of A/T transversion is the least. By in-depth comparative analysis of all raw SNPs (Table S5) and InDels (Table S6) data for strains 891 and 891-B6, only one T/G transversion was identified at the first locus of gene5719 (*chryXb*) in chrysomycin BGC, which was the transcription start site (TSS) and not involved in the biosynthesis of chrysomycins.

## 4. Conclusions

Infectious diseases caused by drug-resistant bacteria have become one of the most serious threats to human health and place the greatest burdens on low-resource settings [25,26]. In order to alleviate this deteriorating situation, it is urgent to search for more effective antibiotics to treat these diseases. As a promising therapeutic agent, CA displayed a potent anti-Gram-positive bacterial effect on drug resistant strains, such as MRSA, MDR-, and XDR-TB, among others. By extensive comparative transcriptome analysis in this work, significant differences were unveiled in two CA-producing isolates, WT strain 891 and its UV-derived mutant 891-B6. The DEGs analysis results showed that 25 genes involved in chrysomycin biosynthesis were up-regulated in mutant 891-B6. Among them, *chryA*, *chryB*, *chryC*, *chryF*, *chryG*, *chryK*, *chryP*, and *chryQ* are responsible for the biosynthesis of benzonaphthopyranone, while *chryD, chryE*, and *chryU* are in charge of the production of deoxyglycoside. Three up-regulated genes *chryOII*, *chryOIII*, and *chryOIV* are conducive to the formation of 8-vinyl in CA from 8-ethyl in CB, which results in higher CA yield and decrease of CB production in mutant 891-B6. The SNPs and InDels analyses displayed that strain 891 has more point mutations and only one T/G transversion is identified at the first locus of gene5719 (*chryXb*) of chrysomycin BGC in mutant 891-B6. These results lay the important foundations for constructing a robust strain for the industrial production of CA and to promote new drug development in the future.

## Figures and Tables

**Table 1:** The up-regulated genes in chrysomycin BGC for mutant 891-B6.

Gene ID	Gene Name	LogFC	*p*-Value	Proposed Function
gene 5694	*chryE*	3.79	0.00	putative TDP-glucose-4,6-dehydratase
gene 5695	*chryD*	4.27	0.00	putative glucose-1-phosphate thymidylyltransferase
gene 5696	*chryXa*	3.00	0.00	unknow
gene 5697	*chryGT*	4.72	0.00	putative C-glycosyltransferase
gene 5698	*chryMT*	4.90	0.00	putative O-methyltransferase
gene 5699	*chryOII*	5.61	0.00	putative anthrone monooxygenase
gene 5700	*chryRM*	5.40	0.00	putative C-methyltransferase
gene 5701	*chryRM*	4.86	0.01	putative C-methyltransferase
gene 5702	*chryU*	-	0.00	putative NDP-hexose 4-ketoreductase
gene 5703	*chryCMT*	5.40	0.00	putative C-methyltransferase
gene 5704	*chryF*	4.41	0.00	putative polyketide ketoreductase
gene 5705	*chryV*	4.71	0.00	hypothetical protein
gene 5706	*chryH*	4.44	0.01	putative oxidoreductase/NADH-dependent FMN reductase
gene 5707	*chryOI*	5.69	0.00	putative FAD-dependent monooxygenase
gene 5708	*chryG*	6.01	0.00	putative cyclase
gene 5710	*chryK*	5.16	0.00	putative bifunctional cyclase/dehydratase
gene 5711	*chryOIV*	5.06	0.00	putative FAD-dependent monooxygenase
gene 5712	*chryA*	4.33	0.00	putative ketoacyl synthase (KS_α_)
gene 5713	*chryB*	8.07	0.00	putative chain length factor (KS_β_)
gene 5714	*chryC*	-	0.01	putative acyl carrier protein
gene 5715	*chryOIII*	5.06	0.00	putative P450 monooxygenase
gene 5717	*chryP*	4.23	0.00	putative malonyl-CoA carrier protein transacylase
gene 5718	*chryQ*	5.37	0.00	putative propionyl-CoA carrier protein transacylase
gene 5723	*chryX1*	4.63	0.02	putative spore-associated protein precursor
gene 5724	-	5.49	0.00	unknown

- No existing.

## Data Availability

The data presented in this study are available in article and supplementary material. The GenBank accession numbers for chromosomal genome and 16S rRNA sequences of *Streptomyces* sp. 891 are PRJNA615006 and CP050693-CP050694, respectively.

## Supplementary Materials

The following supporting information can be downloaded at: https://www.mdpi.com/article/10.3390/metabo12121170/s1, Figure S1: Comparison of HPLC profiles of fermentation extract of two chrysomycin-producing strains *Streptomyces* sp. 891 (a) and 891-B6 (b); Figure S2: Agarose gel electrophoresis of total RNAs of strains 891 (1) and 891-B6 (2); Figure S3: RNA sample quality: image for RNAs HPLC of strain 891 (a) and strain 891-B6 (b); Figure S4: Single base mass distribution of strain 891 (a) and strain B6 (b); Figure S5: The map of RPKM saturation of strains 891 and 891-B6; Table S1: Analysis of RNA-Seq raw data for strains 891 and 891-B6; Table S2: Analysis of RNA-Seq clean data for strains 891 and 891-B6; Table S3: Genome alignment results for strains 891 and 891-B6; Table S4: Results of up- and down-regulated DEGs; Table S5: Raw results of SNPs analysis for strains 891 and 891-B6; Table S6: Raw results of InDels analysis for strains 891 and 891-B6.
