# Supplementary material for: Comparative Transcriptome Analysis of Two Chrysomycin-Producing Wild-Type and Mutant Strains of Streptomyces sp. 891
Source: Metabolites. 2022 Nov 24;12(12):1170. doi: 10.3390/metabo12121170 (PMC9785815; doi:10.3390/metabo12121170)
Supplement: Supplementary file 1 [file metabolites-12-01170-s001.zip › metabolites-2010006-supplementary.pdf]

Supplementary materials for

Comparative Transcriptome Analysis of Two Chrysomycin-Producing Wild-Type and Mutant Strains of *Streptomyces* sp. 891

Wangjie Zhu [1], Xinwei Pei [1], Xiaoyu Chen [1], You Wu [1], Fuhang Song [2] and Huawei Zhang [1,*]

[1] School of Pharmaceutical Sciences, Zhejiang University of Technology, Hangzhou 310014, China
[2] Department of Light Industry, Beijing Technology and Business University, Beijing 100048, China
* Correspondence: hwzhang@zjut.edu.cn; Fax: +86-571-88320913

Contents

Figure S1 Comparison of HPLC profiles of fermentation extract of strains 891 and 891-B6-----------------------2

Figure S2 Agarose gel electrophoresis of total RNAs of strains 891 and 891-B6---3

Figure S3 RNAs HPLC of strain 891 and strain 891-B6--4

Figure S4 Single base mass distribution of strains 891 and 891-B6---5

Figure S5 The map of RPKM saturation of strains 891 and 891-B6---6

Table S1 Analysis of RNA-Seq raw data for strains 891 and 891-B6--7

Table S2 Analysis of RNA-Seq clean data for strains 891 and 891-B6---8

Table S3 Genome alignment results for strains 891 and 891-B6--9

Table S4 Results of up- and down-regulated DEGs--10

Table S5 Raw results of SNPs analysis for strains 891 and 891-B6---22

Table S6 Raw results of InDels analysis for strains 891 and 891-B6--31

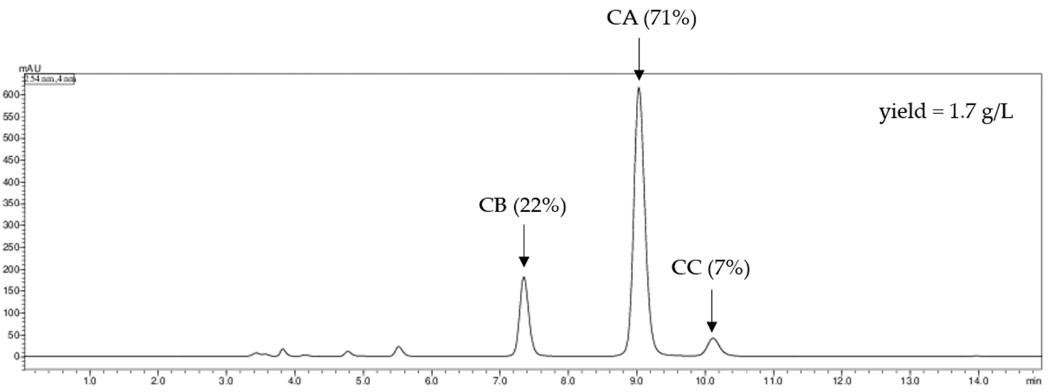

(**a**)

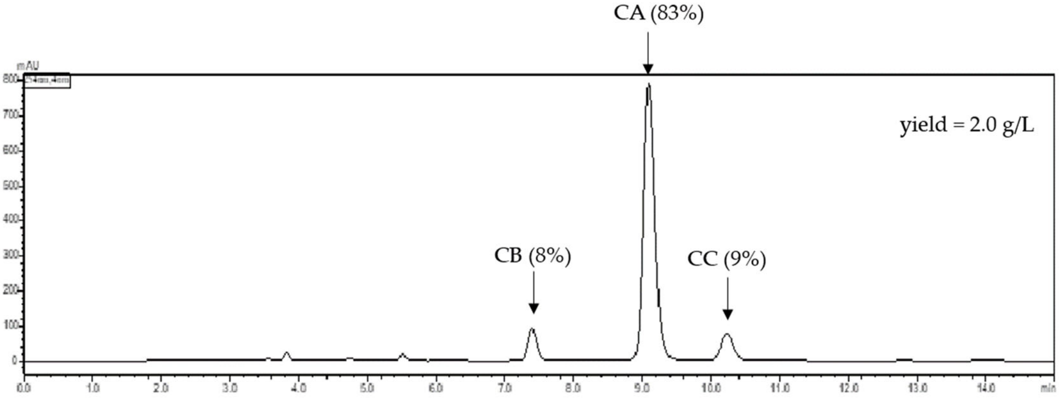

(**b**)

Figure S1. Comparison of HPLC profiles of fermentation extract of strains 891 (**a**) and 891-B6 (**b**). The content of chrysomycins A-C produced by each strain are respectively shown in their upper peaks.

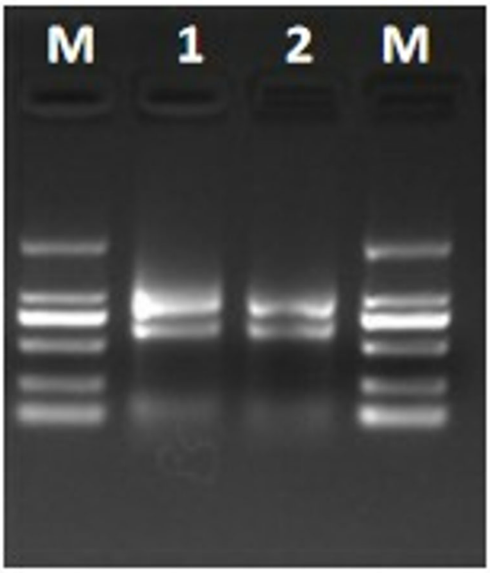

Figure S2. Agarose gel electrophoresis of total RNAs of strains 891 (**1**) and 891-B6 (**2**). The 23S rRNA, 16S rRNA and 5S rRNA strips are clearly visible.

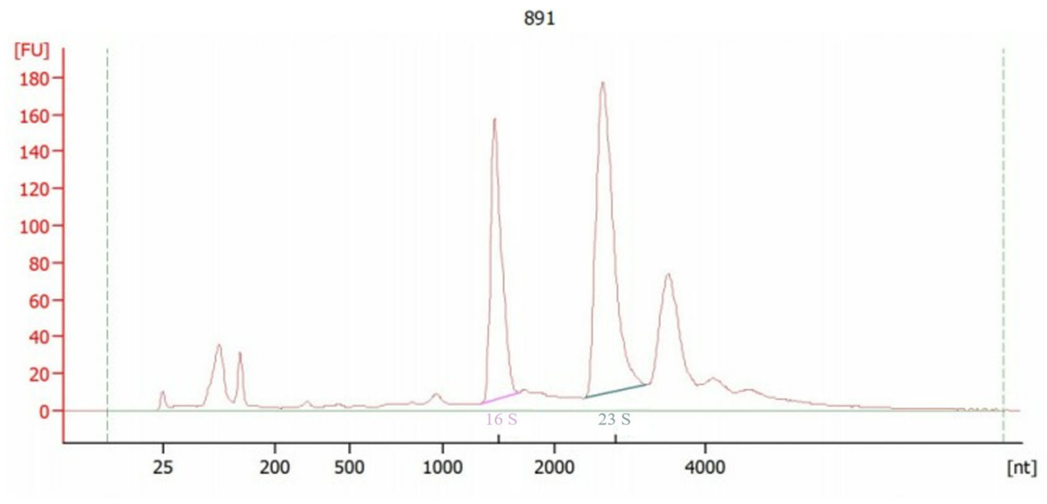

(**a**)

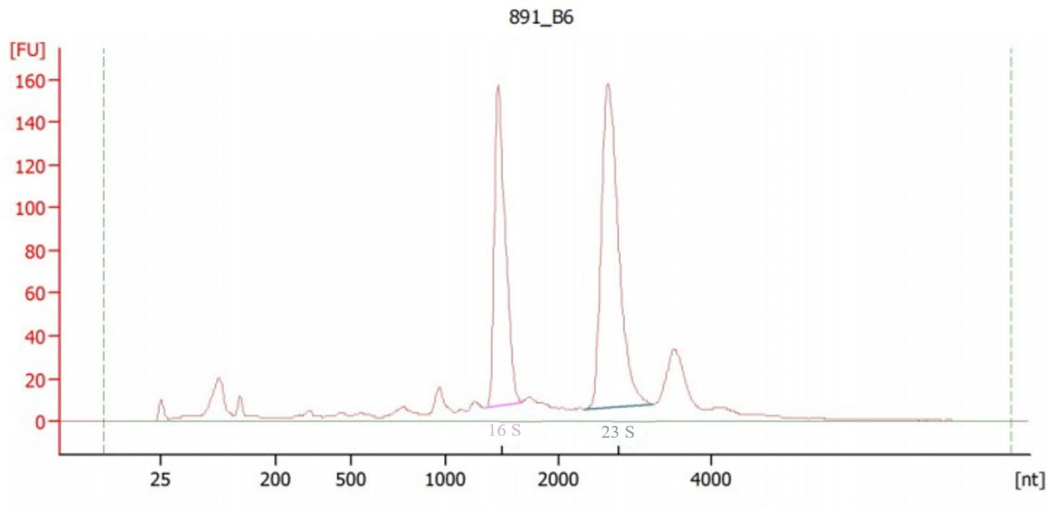

(**b**)

Figure S3. RNA sample quality: images for RNAs HPLC of strain 891 (**a**) and strain 891-B6 (**b**).

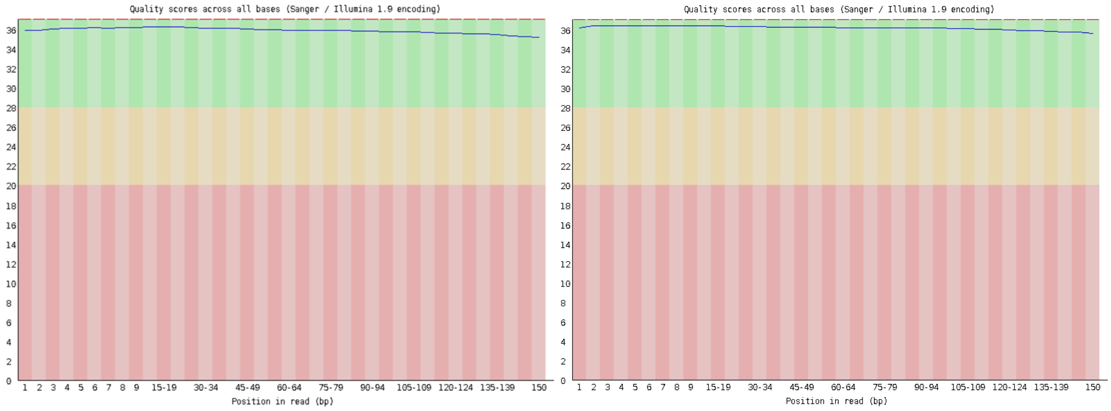

(**a**) (**b**)

Figure S4. Single base mass distribution of strains 891 (**a**) and 891-B6 (**b**).

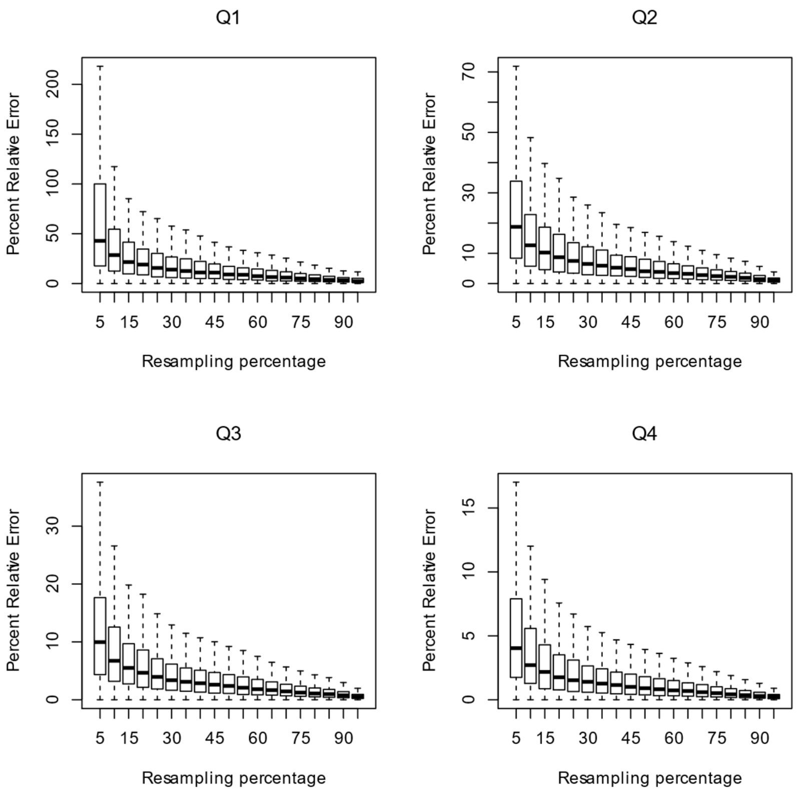

(**a**)

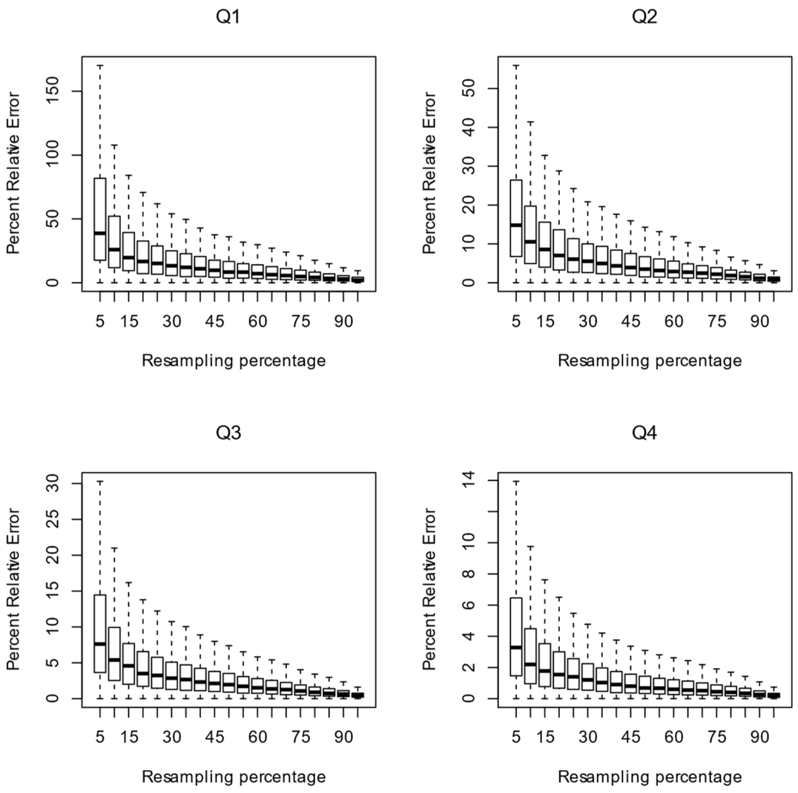

(**b**)

Figure S5. The map of RPKM saturation of strains 891 (**a**) and 891-B6 (**b**). The x-coordinate is the ratio of resampling, and the y-coordinate is the relative error.

Table S1. Analysis of RNA-Seq raw data for strains 891 and 891-B6.

Sample	Reads No.	Bases (bp)	Q30 (bp)	N (%)	Q20 (%)	Q30 (%)
891	34,466,414	5,169,962,100	4,874,820,459	0.00	97.76	94.29
891-B6	35,265,500	5,289,825,000	4,975,382,172	0.00	97.69	94.05

Reads No.: Total number of the read.; Bases (bp): Total number of the bases.; Q30 (bp): The number of bases whose recognition accuracy is above 99.9 %.; N (%): The percentage of fuzzy bases.; Q20 (%): The percentage of bases whose recognition accuracy is above 99 %.; Q30 (%): The percentage of bases whose recognition accuracy is above 99.9 %.

Table S2. Analysis of RNA-Seq clean data for strains 891 and 891-B6.

Sample	Clean Reads No.	Clean Data (bp)	Clean Reads (%)	Clean Data (%)
891	30,717,034	4,607,555,100	89.12	89.12
891-B6	31,907,118	4,786,067,700	90.47	90.47

Clean Reads No.: Number of clean data.; Clean Data (bp): Number of clean bases.; Clean Reads (%): The percentage of clean reads.; Clean Data (%): The percentage of clean bases.

Table S3. Genome alignment results for strains 891 and 891-B6.

Sample	Useful Reads	Total Mapped Reads	(%)	Uniquely Mapped Reads	(%)	Multiple Mapped Reads	(%)
891	30717034	30,171,506	98.22	29,597,879	98.10	573,627	1.90
891-B6	31907118	30,868,643	96.75	30,386,246	98.44	482,397	1.56

Useful Reads: Total number of sequences used for alignment.; Total Mapped Reads (%): Total number and ratio of sequences in the reference genome.; Uniquely Mapped Reads (%): Total number and ratio of sequences aligned to a single position.; Multiple Mapped Reads (%): Total number and ratio of sequences aligned to multiple locations.

Table S4. Results of up- and down-regulated DEGs.

Gene ID	baseMean	log2FoldChange	P-value	Up-regulated/down-regulated
gene348	28399.1169	-Inf	5.9297E-22	down
gene352	61430.0997	-15.1917023	1.1189E-21	down
gene349	13293.0092	-Inf	5.7109E-20	down
gene163	24581.0219	-14.2853053	9.4009E-20	down
gene350	38143.3403	-13.3341616	3.3115E-19	down
gene169	14022.8914	-13.4754898	2.7269E-18	down
gene501	7185.98669	-Inf	2.8955E-18	down
gene281	5530.6814	-Inf	1.6809E-17	down
gene424	5260.78805	-Inf	2.3691E-17	down
gene6585	150535.382	-11.6263886	4.0011E-17	down
gene544	4561.98968	-Inf	6.3847E-17	down
gene6590	31226.0913	-11.6301295	8.6341E-17	down
gene164	8178.25244	-12.28237	1.8647E-16	down
gene421	3862.58208	-Inf	2.0878E-16	down
gene6584	36923.357	-11.2087919	3.1974E-16	down
gene425	2864.03759	-Inf	1.9027E-15	down
gene6588	16869.3489	-10.7413944	3.1526E-15	down
gene6594	49965.175	-10.3848448	5.2964E-15	down
gene6595	56632.9852	-10.278861	7.5018E-15	down
gene426	2208.49527	-Inf	1.4216E-14	down
gene227	2089.69346	-Inf	2.2058E-14	down
gene413	2046.43741	-Inf	2.6072E-14	down
gene527	2044.60969	-Inf	2.626E-14	down
gene165	1902.65676	-Inf	4.689E-14	down
gene6591	3376.22217	-11.0055753	7.3832E-14	down
gene6582	10370.5976	-9.86886454	1.0324E-13	down
gene157	1726.58637	-Inf	1.0366E-13	down
gene6583	5485.64788	-10.1202709	1.4557E-13	down
gene282	1648.60364	-Inf	1.5188E-13	down
gene414	1628.49872	-Inf	1.6816E-13	down
gene354	1623.6248	-Inf	1.724E-13	down
gene166	1551.12523	-Inf	2.5239E-13	down
gene438	1520.66323	-Inf	2.9807E-13	down
gene420	1479.2349	-Inf	3.7608E-13	down
gene458	1445.11746	-Inf	4.5803E-13	down
gene351	1435.36962	-Inf	4.8505E-13	down
gene521	1425.62178	-Inf	5.1389E-13	down
gene510	1416.48318	-Inf	5.4271E-13	down
gene502	1402.47065	-Inf	5.9053E-13	down
gene297	1379.92877	-Inf	6.7785E-13	down
gene355	1375.66409	-Inf	6.9597E-13	down
gene331	1248.94215	-Inf	1.5952E-12	down

gene398	1111.2539	-Inf	4.4106E-12	down
gene519	1085.66581	-Inf	5.412E-12	down
gene167	1309.66727	-11.6396153	5.8852E-12	down
gene526	974.784121	-Inf	1.404E-11	down
gene427	910.204673	-Inf	2.5895E-11	down
gene500	892.53671	-Inf	3.0871E-11	down
gene291	803.587659	-Inf	7.9519E-11	down
gene28	723.777209	-Inf	2.055E-10	down
gene46	698.798366	-Inf	2.8298E-10	down
gene249	679.302684	-Inf	3.6629E-10	down
gene304	640.311319	-Inf	6.2842E-10	down
gene278	617.769436	-Inf	8.7199E-10	down
gene248	601.929194	-Inf	1.1057E-09	down
gene487	592.181353	-Inf	1.2837E-09	down
gene117	568.42099	-Inf	1.8666E-09	down
gene100	564.76555	-Inf	1.98E-09	down
gene162	554.408469	-Inf	2.3449E-09	down
gene518	550.753028	-Inf	2.491E-09	down
gene280	543.442147	-Inf	2.8143E-09	down
gene380	534.303546	-Inf	3.2856E-09	down
gene158	530.038866	-Inf	3.5349E-09	down
gene416	528.820385	-Inf	3.61E-09	down
gene130	512.980143	-Inf	4.7638E-09	down
gene259	482.51814	-Inf	8.3163E-09	down
gene437	469.114858	-Inf	1.074E-08	down
gene252	464.850177	-Inf	1.1667E-08	down
gene279	454.493096	-Inf	1.4308E-08	down
gene343	448.400695	-Inf	1.6166E-08	down
gene112	446.572975	-Inf	1.6774E-08	down
gene300	441.699055	-Inf	1.8522E-08	down
gene383	407.58161	-Inf	3.8167E-08	down
gene337	392.350609	-Inf	5.3642E-08	down
gene460	391.741368	-Inf	5.4391E-08	down
gene525	391.132128	-Inf	5.5151E-08	down
gene171	389.913648	-Inf	5.6706E-08	down
gene253	383.821247	-Inf	6.5239E-08	down
gene330	380.165807	-Inf	7.1029E-08	down
gene70	373.464166	-Inf	8.3167E-08	down
gene260	371.636446	-Inf	8.686E-08	down
gene6596	35207.0628	-5.88363884	1.0384E-07	down
gene461	482.120354	-9.19587434	1.0395E-07	down
gene61	360.670125	-Inf	1.1317E-07	down
gene76	356.405444	-Inf	1.2567E-07	down
gene201	352.750004	-Inf	1.3759E-07	down

gene422	349.703803	-Inf	1.4847E-07	down
gene254	347.266843	-Inf	1.5786E-07	down
gene89	451.04911	-9.09959605	1.8364E-07	down
gene504	401.290317	-9.93210927	1.8723E-07	down
gene269	339.955962	-Inf	1.9015E-07	down
gene415	335.082041	-Inf	2.1567E-07	down
gene471	327.771161	-Inf	2.6125E-07	down
gene246	327.16192	-Inf	2.655E-07	down
gene79	324.72496	-Inf	2.8327E-07	down
gene423	296.699917	-Inf	6.1462E-07	down
gene93	294.872196	-Inf	6.4775E-07	down
gene181	282.687395	-Inf	9.2529E-07	down
gene524	282.078155	-Inf	9.4223E-07	down
gene317	281.468915	-Inf	9.595E-07	down
gene95	279.641195	-Inf	1.0134E-06	down
gene2049	549.62876	7.48728477	1.02E-06	up
gene439	277.813474	-Inf	1.0707E-06	down
gene94	275.376514	-Inf	1.1526E-06	down
gene292	267.456393	-Inf	1.4696E-06	down
gene303	265.019433	-Inf	1.5854E-06	down
gene156	264.410193	-Inf	1.6159E-06	down
gene344	259.536272	-Inf	1.884E-06	down
gene245	252.834631	-Inf	2.3348E-06	down
gene139	251.006911	-Inf	2.4773E-06	down
gene215	251.006911	-Inf	2.4773E-06	down
gene5724	1875.48172	5.49012714	2.66E-06	up
gene277	241.25907	-Inf	3.4159E-06	down
gene2615	18319.3131	-4.92365929	3.6595E-06	down
gene442	238.82211	-Inf	3.7069E-06	down
gene276	238.212869	-Inf	3.7837E-06	down
gene5702	267.956766	Inf	4.13E-06	up
gene256	234.557429	-Inf	4.2831E-06	down
gene195	232.729709	-Inf	4.5593E-06	down
gene69	230.901989	-Inf	4.8549E-06	down
gene2614	8362.18432	-4.88072734	5.2524E-06	down
gene285	225.418828	-Inf	5.8741E-06	down
gene33	224.809588	-Inf	6.001E-06	down
gene228	224.200348	-Inf	6.1308E-06	down
gene5707	962.896611	5.69161602	6.44E-06	up
gene270	221.154147	-Inf	6.8276E-06	down
gene287	219.935667	-Inf	7.13E-06	down
gene5225	22484.8613	-4.72307045	7.182E-06	down
gene2382	11924.1535	-4.74598985	7.4937E-06	down
gene2618	1493.63684	-5.25319783	7.8821E-06	down

gene3107	2415.96769	-5.02025199	7.988E-06	down
gene5713	328.265252	8.06827042	8.16E-06	up
gene18	215.061747	-Inf	8.4934E-06	down
gene301	213.843266	-Inf	8.8769E-06	down
gene473	213.843266	-Inf	8.8769E-06	down
gene375	207.141626	-Inf	1.1352E-05	down
gene247	205.923145	-Inf	1.1877E-05	down
gene160	204.095425	-Inf	1.2716E-05	down
gene5700	918.752876	5.39528899	1.59E-05	up
gene5703	894.343495	5.39833189	1.69E-05	up
gene257	195.566064	-Inf	1.7576E-05	down
gene4114	1078.91183	5.16567255	2.01E-05	up
gene1157	3159.54415	-4.63951355	2.0798E-05	down
gene2616	9153.04075	-4.45137831	2.2226E-05	down
gene217	189.473663	-Inf	2.2271E-05	down
gene283	188.864423	-Inf	2.281E-05	down
gene366	186.427463	-Inf	2.5114E-05	down
gene3108	6702.23123	-4.43233768	2.6415E-05	down
gene4113	4270.01714	4.46801103	2.89E-05	up
gene533	180.335062	-Inf	3.2052E-05	down
gene30	179.725822	-Inf	3.2853E-05	down
gene178	179.116582	-Inf	3.3675E-05	down
gene2617	6105.23667	-4.36414743	3.4478E-05	down
gene255	178.507342	-Inf	3.452E-05	down
gene212	176.679622	-Inf	3.7194E-05	down
gene29	176.070382	-Inf	3.8134E-05	down
gene316	175.461142	-Inf	3.91E-05	down
gene60	173.633421	-Inf	4.2162E-05	down
gene5699	548.110894	5.61415888	4.39E-05	up
gene229	171.196461	-Inf	4.6654E-05	down
gene509	171.196461	-Inf	4.6654E-05	down
gene381	170.587221	-Inf	4.7856E-05	down
gene182	166.931781	-Inf	5.5811E-05	down
gene5710	715.444648	5.15775496	5.63E-05	up
gene92	162.05786	-Inf	6.872E-05	down
gene5049	407.610921	5.90261764	7.21E-05	up
gene4116	1730.23972	4.39338031	8.32E-05	up
gene318	154.746979	-Inf	9.4525E-05	down
gene5715	626.001505	5.05529983	0.0001036	up
gene470	152.310019	-Inf	0.00010532	down
gene455	151.091539	-Inf	0.00011121	down
gene472	150.482299	-Inf	0.00011428	down
gene6597	551.724462	-5.1035977	0.00012397	down
gene523	148.654578	-Inf	0.00012408	down

gene6257	1542.75289	-4.28868434	0.00013036	down
gene382	147.436098	-Inf	0.00013111	down
gene459	147.436098	-Inf	0.00013111	down
gene236	145.608378	-Inf	0.00014248	down
gene2619	5657.70063	-3.93339782	0.00014604	down
gene243	143.171418	-Inf	0.00015933	down
gene251	143.171418	-Inf	0.00015933	down
gene6225	660.830776	4.78367778	0.00017078	up
gene357	140.734457	-Inf	0.00017834	down
gene1970	5450.58413	-3.86512016	0.0001845	down
gene295	138.906737	-Inf	0.0001942	down
gene103	137.688257	-Inf	0.00020562	down
gene3296	3022.94457	-3.90831083	0.0002177	down
gene308	136.469777	-Inf	0.00021776	down
gene328	135.860537	-Inf	0.00022412	down
gene4762	2262.47897	3.96149445	0.00022849	up
gene4115	731.546591	4.55490527	0.00023432	up
gene741	4942.89872	-3.79784101	0.00023765	down
gene3785	3834.53191	3.81324942	0.00025107	up
gene250	132.814336	-Inf	0.00025907	down
gene522	132.205096	-Inf	0.00026675	down
gene71	132.205096	-Inf	0.00026675	down
gene144	131.595856	-Inf	0.00027466	down
gene397	131.595856	-Inf	0.00027466	down
gene2611	5708.99404	-3.73480348	0.00027479	down
gene2622	7214.58747	-3.70394837	0.00028174	down
gene5697	566.476028	4.71958219	0.00029263	up
gene5360	1490.07351	-4.01154374	0.00030256	down
gene404	129.158896	-Inf	0.00030896	down
gene5359	695.245513	-4.47309808	0.00030983	down
gene4763	954.997427	4.23588441	0.00031295	up
gene2610	4313.87405	-3.72526443	0.00031654	down
gene5704	744.229671	4.406698	0.00032529	up
gene3783	2358.69703	3.8079697	0.00034974	up
gene5080	189.978223	7.27532455	0.0003584	up
gene5712	759.797744	4.3318263	0.00037516	up
gene2789	1371.84325	-3.95127153	0.00039611	down
gene540	123.066495	-Inf	0.00041663	down
gene3194	53744.9109	-3.47427798	0.00046525	down
gene2609	3661.69825	-3.62275788	0.00046943	down
gene240	120.629535	-Inf	0.00047048	down
gene384	120.020295	-Inf	0.00048508	down
gene226	119.411055	-Inf	0.00050017	down
gene5708	239.194772	6.00977893	0.0005185	up

gene772	471.329896	-4.64989269	0.0005237	down
gene5226	1331.65853	-3.86069149	0.00052837	down
gene11	118.192575	-Inf	0.00053189	down
gene2621	1852.60067	-3.73195116	0.00053905	down
gene5711	355.048786	5.05661718	0.00056512	up
gene503	116.974094	-Inf	0.00056579	down
gene233	116.364854	-Inf	0.00058361	down
gene378	116.364854	-Inf	0.00058361	down
gene123	115.755614	-Inf	0.00060203	down
gene154	115.146374	-Inf	0.00062108	down
gene2620	1437.59303	-3.76525057	0.00063099	down
gene5717	649.650907	4.22857629	0.0006619	up
gene2612	530.488991	-4.38622712	0.00069686	down
gene6747	1925.06465	-3.60618472	0.00074883	down
gene146	110.881694	-Inf	0.00077402	down
gene469	110.881694	-Inf	0.00077402	down
gene5109	400.136739	4.65942242	0.00084258	up
gene5695	555.532736	4.26777774	0.00084602	up
gene315	109.053973	-Inf	0.00085157	down
gene4519	50587.5336	-3.24534006	0.0009675	down
gene5718	257.224928	5.36529425	0.00099151	up
gene258	105.398533	-Inf	0.00103292	down
gene3784	467.980122	4.34604573	0.00106164	up
gene771	6248.01658	-3.28135015	0.00109535	down
gene155	104.180053	-Inf	0.00110227	down
gene96	104.180053	-Inf	0.00110227	down
gene4245	18921.4977	-3.21181035	0.00113637	down
gene5110	378.798681	4.5771412	0.00114779	up
gene5337	793.693266	3.84062253	0.00116338	up
gene128	102.961573	-Inf	0.00117665	down
gene237	101.743093	-Inf	0.00125646	down
gene5698	299.863359	4.89540091	0.00127187	up
gene5705	330.203934	4.70595891	0.00132765	up
gene2211	2290.98764	-3.36161126	0.001328	down
gene24	100.524612	-Inf	0.00134211	down
gene2788	466.108436	-4.21601024	0.001358	down
gene4761	1325.3484	3.51487061	0.00136442	up
gene520	97.4784121	-Inf	0.00158493	down
gene153	96.2599319	-Inf	0.00169493	down
gene2613	1445.18027	-3.39359518	0.00173849	down
gene2932	4101.75801	-3.15966215	0.00179306	down
gene2636	1718.64532	-3.31258268	0.00186981	down
gene119	94.4322117	-Inf	0.00187558	down
gene19	94.4322117	-Inf	0.00187558	down

gene4759	11817.8192	3.05215649	0.0019194	up
gene4243	92234.6304	-3.01964418	0.00192053	down
gene508	93.8229716	-Inf	0.00194031	down
gene5066	102.997164	Inf	0.00209657	up
gene4242	74520.8789	-2.98360515	0.00215204	down
gene5048	261.502171	4.78738651	0.00229278	up
gene5050	366.251685	4.25086436	0.00232313	up
gene5696	8278.12594	3.00509858	0.00233317	up
gene125	89.5582911	-Inf	0.00246638	down
gene323	88.949051	-Inf	0.00255326	down
gene62	88.949051	-Inf	0.00255326	down
gene84	88.3398109	-Inf	0.00264344	down
gene430	87.1213308	-Inf	0.0028342	down
gene372	86.5120907	-Inf	0.00293508	down
gene457	86.5120907	-Inf	0.00293508	down
gene1143	3132.76062	-3.02362322	0.00294424	down
gene216	85.9028506	-Inf	0.00303982	down
gene2019	4749.66703	-2.96138707	0.00304226	down
gene190	85.2936105	-Inf	0.00314858	down
gene951	2315.72362	-2.98976849	0.00373078	down
gene306	81.6381701	-Inf	0.0038953	down
gene4241	21421.4107	-2.79435864	0.00396689	down
gene235	81.02893	-Inf	0.00403725	down
gene505	81.02893	-Inf	0.00403725	down
gene543	81.02893	-Inf	0.00403725	down
gene2786	1662.23722	-3.02574321	0.00414344	down
gene2175	1154.08703	-3.137426	0.00417873	down
gene26	80.4196899	-Inf	0.00418475	down
gene542	79.8104499	-Inf	0.00433804	down
gene1317	3573.77297	-2.85123383	0.00453792	down
gene5694	398.581187	3.79377012	0.00459155	up
gene126	77.9827296	-Inf	0.00483516	down
gene67	77.9827296	-Inf	0.00483516	down
gene14	77.3734896	-Inf	0.00501416	down
gene365	77.3734896	-Inf	0.00501416	down
gene497	76.7642495	-Inf	0.00520028	down
gene73	76.7642495	-Inf	0.00520028	down
gene108	76.1550094	-Inf	0.00539382	down
gene34	76.1550094	-Inf	0.00539382	down
gene4723	5562.39329	2.74237343	0.00539618	up
gene6071	1902.34481	-2.87539071	0.00564859	down
gene102	74.3272892	-Inf	0.00602222	down
gene213	73.7180491	-Inf	0.00624875	down
gene2634	2811.05883	-2.76414351	0.00628926	down

gene468	73.108809	-Inf	0.00648444	down
gene4240	35409.7492	-2.61598223	0.0064855	down
gene5105	211.029457	4.46545841	0.00675221	up
gene143	71.8903289	-Inf	0.00698486	down
gene234	71.8903289	-Inf	0.00698486	down
gene441	71.8903289	-Inf	0.00698486	down
gene3969	8456.74843	-2.62020476	0.00712437	down
gene4376	73726.166	-2.5730509	0.00720015	down
gene211	71.2810888	-Inf	0.00725044	down
gene88	71.2810888	-Inf	0.00725044	down
gene339	70.6718487	-Inf	0.00752686	down
gene2893	1093.67387	-2.9150304	0.007589	down
gene963	1730.25228	-2.77961886	0.00764757	down
gene6070	13866.5239	-2.5757276	0.00764915	down
gene4361	7857.18711	-2.5938297	0.007744	down
gene3971	4080.15197	-2.63955521	0.00778757	down
gene152	70.0626087	-Inf	0.00781459	down
gene242	70.0626087	-Inf	0.00781459	down
gene773	333.704447	-3.67620714	0.00792629	down
gene4751	1058.10136	-2.90343857	0.0080307	down
gene147	69.4533686	-Inf	0.00811413	down
gene17	69.4533686	-Inf	0.00811413	down
gene368	69.4533686	-Inf	0.00811413	down
gene3763	6338.25529	-2.58007315	0.00831977	down
gene975	765.450487	-3.02573448	0.00832695	down
gene151	68.8441285	-Inf	0.00842599	down
gene225	68.8441285	-Inf	0.00842599	down
gene286	68.8441285	-Inf	0.00842599	down
gene5099	195.237368	4.45220231	0.00843387	up
gene5801	500.054205	-3.2764343	0.00846244	down
gene5131	1323.30294	2.80436261	0.00854382	up
gene2274	2225.36138	-2.68457816	0.00857121	down
gene310	68.2348884	-Inf	0.00875071	down
gene332	68.2348884	-Inf	0.00875071	down
gene729	1757.38335	-2.71807729	0.00886386	down
gene5098	289.504184	3.79177828	0.00891899	up
gene3252	1476.30804	-2.75210588	0.00903658	down
gene293	67.6256484	-Inf	0.00908885	down
gene4722	7334.01109	2.52299445	0.00942513	up
gene180	67.0164083	-Inf	0.00944102	down
gene3157	1448.66822	-2.73869592	0.00945669	down
gene2000	190.3132	4.41364247	0.00948001	up
gene3970	2598.88208	-2.61451044	0.00963155	down
gene1347	1073.00995	-2.82183784	0.00964022	down

gene6066	296.714572	-3.68990474	0.00976584	down
gene4150	3545.55741	-2.55429728	0.01016571	down
gene4760	2265.92924	2.60829979	0.01030503	up
gene587	2495.82001	-2.59250765	0.01035809	down
gene5706	180.265973	4.4436403	0.0104832	up
gene2771	4792.48133	-2.50916031	0.01063196	down
gene136	64.579448	-Inf	0.01100269	down
gene5701	146.033381	4.85609926	0.01137252	up
gene179	63.9702079	-Inf	0.01143484	down
gene185	63.9702079	-Inf	0.01143484	down
gene296	63.9702079	-Inf	0.01143484	down
gene5242	8646.03995	-2.44138815	0.01158902	down
gene811	1023.64894	-2.75943585	0.01160483	down
gene106	63.3609678	-Inf	0.01188519	down
gene127	63.3609678	-Inf	0.01188519	down
gene32	63.3609678	-Inf	0.01188519	down
gene5751	2931.07494	-2.50160866	0.01235838	down
gene591	1005.5832	-2.71959804	0.01291264	down
gene894	3328.20735	-2.46463238	0.01307731	down
gene161	61.5332476	-Inf	0.01335378	down
gene4112	530.796752	3.01354412	0.01340896	up
gene5475	48680.0365	-2.34338914	0.01370314	down
gene5714	67.7072991	Inf	0.0137789	up
gene514	60.9240075	-Inf	0.01388547	down
gene64	60.9240075	-Inf	0.01388547	down
gene47	60.3147675	-Inf	0.01443986	down
gene2272	14840.3366	-2.33562929	0.01465542	down
gene1111	6670.47119	-2.36405037	0.01472151	down
gene436	59.7055274	-Inf	0.01501796	down
gene3388	676.911783	-2.81698139	0.01503102	down
gene5545	1552.6431	2.52604982	0.0152227	up
gene562	303.466459	-3.39988823	0.01526287	down
gene588	1644.20288	-2.51107075	0.01533677	down
gene3879	12328.5822	2.31816707	0.01545993	up
gene3139	9447.03991	-2.32871123	0.01548977	down
gene492	59.0962873	-Inf	0.01562085	down
gene541	59.0962873	-Inf	0.01562085	down
gene2243	1933.69659	-2.47116644	0.01564052	down
gene5723	141.308106	4.63059691	0.01578931	up
gene5542	1838.72423	2.46059676	0.01638184	up
gene2244	5669.03962	-2.32835676	0.01656034	down
gene373	57.8778072	-Inf	0.0169056	down
gene6057	938.554225	-2.62001405	0.01710622	down
gene5032	160.569304	4.26855359	0.01726071	up

gene515	57.2685671	-Inf	0.01758989	down
gene4966	4266.20677	2.31612723	0.01792584	up
gene5343	938.169001	-2.59328434	0.01816181	down
gene338	56.659327	-Inf	0.01830383	down
gene5453	485.334043	-2.90860531	0.01832001	down
gene3133	31968.0348	-2.22556906	0.01894235	down
gene2608	1023.42702	-2.54424215	0.01898677	down
gene20	56.0500869	-Inf	0.0190488	down
gene1672	2055.92146	-2.37446386	0.01929262	down
gene116	55.4408469	-Inf	0.01982622	down
gene222	55.4408469	-Inf	0.01982622	down
gene419	55.4408469	-Inf	0.01982622	down
gene5486	142252.587	-2.19730482	0.0199515	down
gene6573	1414.85435	2.43231634	0.02000732	up
gene342	54.8316068	-Inf	0.02063761	down
gene1462	332.58646	-3.13176401	0.02067047	down
gene5400	1938.99552	-2.35413575	0.02074375	down
gene5440	881.934677	2.53511595	0.02170962	up
gene3383	6986.45952	-2.20701733	0.02193769	down
gene5078	344.314855	3.07369069	0.02200329	up
gene173	53.6131266	-Inf	0.02236863	down
gene5100	164.859108	3.99207693	0.02257068	up
gene2554	1022.89315	-2.46165576	0.02282992	down
gene188	53.0038866	-Inf	0.02329166	down
gene5550	2926.85422	2.23645404	0.02394441	up
gene488	52.3946465	-Inf	0.02425544	down
gene5264	34214.1564	-2.1271458	0.02439876	down
gene2183	5948.5217	-2.16437433	0.02493498	down
gene294	51.7854064	-Inf	0.02526186	down
gene5535	1123.24609	-2.38827813	0.02528231	down
gene6589	2284.04523	-2.24256171	0.02548515	down
gene1034	717.745523	-2.53752074	0.02551636	down
gene517	51.1761663	-Inf	0.02631293	down
gene4586	474.828316	-2.72525001	0.02665528	down
gene3138	70759.4851	-2.07876207	0.02725386	down
gene3097	1061.83636	-2.36767263	0.02739284	down
gene239	50.5669263	-Inf	0.02741077	down
gene41	50.5669263	-Inf	0.02741077	down
gene477	50.5669263	-Inf	0.02741077	down
gene214	49.9576862	-Inf	0.02855756	down
gene6	49.9576862	-Inf	0.02855756	down
gene3490	2153.72857	-2.202086	0.02859801	down
gene6005	1829.18366	-2.22382652	0.02884817	down
gene5107	175.701907	3.67051228	0.02932196	up

gene379	49.3484461	-Inf	0.02975564	down
gene536	49.3484461	-Inf	0.02975564	down
gene6289	1433.64239	-2.25254596	0.03000861	down
gene5034	129.805821	4.19922157	0.03021579	up
gene3453	2196.57217	2.16866823	0.03068656	up
gene196	48.739206	-Inf	0.03100742	down
gene448	48.739206	-Inf	0.03100742	down
gene2951	832.981923	-2.38066835	0.03138248	down
gene2323	1391.76603	-2.22981426	0.03202165	down
gene5222	2226.21558	2.14715954	0.03212529	up
gene371	48.129966	-Inf	0.03231547	down
gene435	48.129966	-Inf	0.03231547	down
gene1599	4687.77368	-2.06706418	0.03277966	down
gene5543	4718.42621	2.06365835	0.03294446	up
gene749	192.484272	-3.42316309	0.03301727	down
gene5456	41778.7847	-2.00484464	0.03306904	down
gene6770	1327.48388	-2.22511552	0.03310383	down
gene2577	245.745673	3.14374486	0.03310645	up
gene403	47.5207259	-Inf	0.03368245	down
gene1971	455.146302	-2.61119277	0.03445808	down
gene1955	3430.28961	-2.06106601	0.03518739	down
gene3186	1083.12417	-2.2443117	0.03534677	down
gene3641	787.251232	-2.33142401	0.03617036	down
gene1020	2643.59527	-2.07314599	0.03634029	down
gene80	46.3022457	-Inf	0.03660464	down
gene90	46.3022457	-Inf	0.03660464	down
gene5544	2803.23711	2.06000958	0.03687849	up
gene341	45.6930056	-Inf	0.0381659	down
gene511	45.0837656	-Inf	0.03979823	down
gene1316	320.477028	-2.7511578	0.04088276	down
gene2118	1835.19231	-2.06428293	0.04137265	down
gene5518	1069.61831	2.17109014	0.04165304	up
gene2702	311.325866	-2.75311718	0.04226852	down
gene284	43.8652854	-Inf	0.04328997	down
gene453	43.8652854	-Inf	0.04328997	down
gene2964	6126.72546	-1.92924659	0.04390219	down
gene4994	10191.435	1.9077767	0.04411052	up
gene5762	1226.52379	-2.10736665	0.04436371	down
gene4965	20373.6267	1.88961671	0.0445185	up
gene131	43.2560453	-Inf	0.04515675	down
gene220	43.2560453	-Inf	0.04515675	down
gene5124	573.242571	-2.34306095	0.04528458	down
gene4321	3222.71251	-1.95645002	0.04542618	down
gene2210	271.252105	-2.81994381	0.04557249	down

gene986	178.882098	-3.26789593	0.0456168	down
gene176	42.6468053	-Inf	0.04710936	down
gene97	42.6468053	-Inf	0.04710936	down
gene4906	205.129668	-3.07586472	0.0471591	down
gene3146	339.426279	-2.61606635	0.04741422	down
gene6020	5333.15363	1.89986124	0.04773891	up
gene5632	19443.6897	-1.8515358	0.04889397	down
gene145	42.0375652	-Inf	0.04915198	down
gene175	42.0375652	-Inf	0.04915198	down
gene5401	776.994644	-2.18160021	0.04922068	down
gene3225	2272.75021	-1.9559918	0.04931006	down
gene2328	1815.62336	-1.98156748	0.04980511	down
gene1436	6518.11929	-1.87112655	0.04994623	down

baseMean: the homogenization of the read count of this gene in two samples for differentially expression.

Table S5. Raw results of SNPs analysis for strains 891 and 891-B6.

CHROM	POS	REF/ALT	891	891-B6	GENE_ID
chr	42539	A/G	15/8	0/0	gene49
chr	91084	C/G	154/56	0/0	gene89
chr	91085	T/G	152/57	0/0	gene89
chr	93213	G/C	13/6	0/0	gene91
chr	107802	T/C	24/7	0/0	-
chr	204252	C/A	22/8	0/0	gene197
chr	298731	T/C	17/7	0/0	-
chr	603769	A/G	19/7	0/0	gene545
chr	706472	G/A	2/6	8/0	gene628
chr	764890	T/C	0/14	0/0	-
chr	777091	A/T	0/8	0/0	gene687
chr	815640	T/G	14/7	9/2	-
chr	852389	C/T	0/103	421/0	gene762
chr	866037	G/C	27/14	0/0	gene773
chr	866047	A/T	7/6	0/0	gene773
chr	872075	C/T	8/7	20/0	gene776
chr	920465	G/T	20/8	15/0	gene823
chr	1045224	T/C	8/8	0/0	gene933
chr	1047291	G/A	2/6	8/0	gene935
chr	1085388	T/G	175/27	23/8	gene975
chr	1135154	G/C	152/45	181/86	gene1013
chr	1149175	G/C	16/7	48/17	gene1023
chr	1257013	G/C	20/0	0/31	gene1112
chr	1257014	G/A	20/0	0/31	gene1112
chr	1258886	G/A	17/1	0/44	gene1115
chr	1258887	G/A	17/0	0/42	gene1115
chr	1311449	C/T	20/0	0/18	gene1163
chr	1371213	C/T	34/9	22/0	gene1205
chr	1383161	C/T	35/9	61/0	gene1209
chr	1413469	G/C	55/12	62/17	gene1209
chr	1415053	G/A	40/0	0/153	gene1209
chr	1439463	A/C	16/7	45/5	gene1209
chr	1469238	T/G	7/6	11/2	gene1221
chr	1469845	G/A	19/9	29/0	gene1221
chr	1588988	T/A	32/8	14/5	gene1327
chr	1624069	G/A	11/9	24/0	gene1359
chr	1640040	A/C	19/7	51/6	gene1372
chr	1640054	T/G	26/7	61/4	gene1372
chr	1671913	T/G	65/17	82/24	gene1404
chr	1704689	C/T	165/0	0/147	gene1431
chr	1719677	G/A	2021/0	0/2184	gene1441
chr	1728687	A/C	2540/612	1394/422	gene1449
chr	1767755	T/G	25/4	59/34	gene1484
chr	1817915	G/C	212/0	0/352	gene1530
chr	1830554	A/T	57/1	40/10	gene1544
chr	1830575	T/G	56/19	54/16	gene1544
chr	1830584	A/C	66/9	51/20	gene1544
chr	1884085	T/G	75/26	192/26	gene1581
chr	1884098	G/T	70/30	163/26	gene1581
chr	1922671	T/G	13/6	33/0	gene1611
chr	1946319	C/A	100/179	72/400	chr.rRNA2
chr	1946336	C/T	92/166	74/386	chr.rRNA2
chr	1946361	A/T	91/130	62/272	chr.rRNA2
chr	1946362	T/C	89/133	66/277	chr.rRNA2
chr	1946372	A/G	91/136	61/277	chr.rRNA2
chr	1946386	A/G	100/103	63/213	chr.rRNA2
chr	1946391	C/A	110/97	70/205	chr.rRNA2
chr	1946404	C/A	202/91	153/192	chr.rRNA2

chr	1946406	G/A	224/89	159/188	chr.rRNA2
chr	1946412	C/G	233/87	164/186	chr.rRNA2
chr	1946431	C/T	258/59	183/137	chr.rRNA2
chr	1946435	G/C	259/55	180/119	chr.rRNA2
chr	1946442	C/T	259/25	186/55	chr.rRNA2
chr	1946444	A/C	262/21	179/55	chr.rRNA2
chr	1946446	C/G	263/24	184/53	chr.rRNA2
chr	1946447	C/T	265/25	180/58	chr.rRNA2
chr	1946451	G/A	263/26	172/54	chr.rRNA2
chr	1947122	C/A	809/489	485/330	chr.rRNA2
chr	1947123	C/G	808/470	492/319	chr.rRNA2
chr	1947124	A/G	786/492	477/333	chr.rRNA2
chr	1947129	T/A	763/477	461/327	chr.rRNA2
chr	1947132	T/C	799/489	485/328	chr.rRNA2
chr	1947133	G/C	771/491	469/327	chr.rRNA2
chr	1947134	G/C	794/493	481/331	chr.rRNA2
chr	1947476	T/C	738/618	695/483	chr.rRNA2
chr	1947478	C/G	745/623	710/481	chr.rRNA2
chr	1947479	C/T	732/622	702/474	chr.rRNA2
chr	1947480	A/C	686/620	674/487	chr.rRNA2
chr	1947481	C/T	732/613	709/471	chr.rRNA2
chr	1947490	G/A	720/593	702/455	chr.rRNA2
chr	1947491	C/A	718/592	711/453	chr.rRNA2
chr	1947493	G/C	724/598	709/454	chr.rRNA2
chr	1947495	A/G	703/596	691/461	chr.rRNA2
chr	1947496	G/A	725/600	705/444	chr.rRNA2
chr	1947771	C/A	89/376	59/316	chr.rRNA2
chr	1947772	A/C	58/420	37/359	chr.rRNA2
chr	1947773	C/A	59/394	41/334	chr.rRNA2
chr	1947777	C/G	87/387	58/328	chr.rRNA2
chr	1947778	C/G	60/399	38/339	chr.rRNA2
chr	1947785	A/C	57/349	40/328	chr.rRNA2
chr	1947786	G/C	80/352	54/324	chr.rRNA2
chr	1947789	A/C	80/352	55/329	chr.rRNA2
chr	1947790	T/A	54/367	39/331	chr.rRNA2
chr	1947791	T/C	75/350	56/320	chr.rRNA2
chr	1947794	G/T	65/376	48/343	chr.rRNA2
chr	1947795	T/G	64/378	47/341	chr.rRNA2
chr	1947796	G/C	93/354	61/329	chr.rRNA2
chr	1948418	A/C	311/253	191/170	chr.rRNA2
chr	1948420	A/C	485/147	275/91	chr.rRNA2
chr	1948421	C/G	490/249	273/173	chr.rRNA2
chr	1948423	G/T	284/249	178/169	chr.rRNA2
chr	1948424	T/C	466/252	269/171	chr.rRNA2
chr	1948425	G/C	465/245	264/172	chr.rRNA2
chr	1948427	G/A	538/177	360/83	chr.rRNA2
chr	1948429	A/G	518/174	357/81	chr.rRNA2
chr	1948430	C/G	463/244	269/178	chr.rRNA2
chr	1948432	C/A	289/250	188/178	chr.rRNA2
chr	1948433	T/C	471/251	271/178	chr.rRNA2
chr	1948434	G/C	453/253	264/177	chr.rRNA2
chr	1948435	C/A	285/249	186/175	chr.rRNA2
chr	1948437	T/G	449/244	262/176	chr.rRNA2
chr	1948617	C/A	2165/668	2473/432	chr.rRNA2
chr	1948618	A/C	2167/666	2457/424	chr.rRNA2
chr	1948619	A/C	2156/668	2460/425	chr.rRNA2
chr	1948625	G/A	2064/648	2372/410	chr.rRNA2
chr	1948636	A/T	2110/555	2419/369	chr.rRNA2
chr	1948695	T/C	2594/928	2635/508	chr.rRNA2
chr	1948696	C/A	2594/919	2634/504	chr.rRNA2
chr	1948698	A/C	2619/920	2640/498	chr.rRNA2

chr	1948699	C/A	2649/904	2656/494	chr.rRNA2
chr	1948702	G/A	2650/920	2686/503	chr.rRNA2
chr	1948703	G/A	2643/915	2697/493	chr.rRNA2
chr	1948709	A/T	2650/1013	2655/552	chr.rRNA2
chr	1948714	A/G	2622/1027	2633/577	chr.rRNA2
chr	1948715	C/T	2628/1022	2650/557	chr.rRNA2
chr	1948721	C/A	2578/1044	2607/576	chr.rRNA2
chr	1948732	G/C	2578/1065	2588/602	chr.rRNA2
chr	1948740	C/T	2602/1075	2591/601	chr.rRNA2
chr	1948742	C/A	2604/1086	2597/608	chr.rRNA2
chr	1948743	A/G	2596/1087	2580/608	chr.rRNA2
chr	1948744	G/C	2650/1086	2589/605	chr.rRNA2
chr	1949127	A/C	27/9	30/4	-
chr	1949131	C/A	32/8	35/4	-
chr	1949134	A/C	26/8	31/4	-
chr	1949137	C/G	31/8	35/4	-
chr	1949138	T/C	30/8	34/4	-
chr	1949177	G/A	3/7	14/3	-
chr	1950085	C/T	513/282	220/123	chr.rRNA3
chr	1950705	G/T	1918/933	931/441	gene1625,
chr	1950706	A/G	1921/940	920/446	gene1625,
chr	1950707	C/A	1922/938	936/448	gene1625,
chr	1950708	C/G	1914/943	927/447	gene1625,
chr	1950713	G/C	1895/918	938/434	gene1625,
chr	1950714	G/T	1911/919	945/438	gene1625,
chr	1950716	C/A	1924/905	945/438	gene1625,
chr	1950717	G/A	1914/909	947/435	gene1625,
chr	1950724	A/G	1967/1006	981/476	gene1625,
chr	1992994	C/G	12/6	13/0	-
chr	2014626	T/C	22/7	44/0	gene1670
chr	2014812	A/G	22/7	21/0	gene1670
chr	2014830	G/A	25/0	23/7	gene1670
chr	2041992	A/G	0/92	164/0	gene1691
chr	2047666	T/C	20/9	14/0	-
chr	2092530	C/G	23/7	15/3	gene1735
chr	2092531	C/G	21/8	15/5	gene1735
chr	2092532	T/G	22/8	16/4	gene1735
chr	2111917	T/G	0/0	11/6	gene1756
chr	2131462	A/G	38/4	67/18	gene1775
chr	2287495	C/G	0/0	8/6	-
chr	2303710	A/G	14/7	21/0	gene1918
chr	2310312	C/T	2/6	0/0	gene1924
chr	2364244	C/G	22/14	22/19	gene1978
chr	2364248	T/G	21/17	18/22	gene1978
chr	2429500	A/G	0/42	27/0	gene2037
chr	2546973	T/G	8/0	10/7	-
chr	2667032	T/G	0/0	8/8	gene2248
chr	2696320	C/G	64/22	67/13	gene2277
chr	2722741	G/C	44/50	64/44	gene2304
chr	2793384	G/T	2/6	0/0	gene2370
chr	2817618	G/C	88/61	112/42	gene2392
chr	2999371	T/G	2285/71	691/186	gene2567
chr	3015076	T/G	12/10	12/19	-
chr	3015078	T/G	20/11	31/20	-
chr	3015083	T/G	19/23	16/49	-
chr	3015089	A/C	11/13	19/20	-
chr	3031470	C/G	23/2	27/13	-
chr	3082633	G/C	65/93	99/60	gene2644
chr	3111926	A/G	49/15	109/1	gene2672
chr	3115067	T/C	25/18	67/41	gene2675
chr	3115524	G/A	112/191	130/191	gene2675

chr	3115613	A/G	164/54	177/82	gene2675
chr	3140811	C/A	103/198	65/427	chr.rRNA5
chr	3140828	C/T	107/188	58/417	chr.rRNA5
chr	3140853	A/T	107/138	57/289	chr.rRNA5
chr	3140854	T/C	107/139	65/285	chr.rRNA5
chr	3140864	A/G	111/140	51/287	chr.rRNA5
chr	3140878	A/G	107/118	57/214	chr.rRNA5
chr	3140883	C/A	118/109	60/204	chr.rRNA5
chr	3140896	C/A	199/96	126/175	chr.rRNA5
chr	3140898	G/A	212/101	138/175	chr.rRNA5
chr	3140904	C/G	234/101	154/181	chr.rRNA5
chr	3140923	C/T	264/79	165/135	chr.rRNA5
chr	3140927	G/C	262/74	168/116	chr.rRNA5
chr	3140934	C/T	268/26	171/69	chr.rRNA5
chr	3140936	A/C	266/26	167/68	chr.rRNA5
chr	3140938	C/G	264/26	166/66	chr.rRNA5
chr	3140939	C/T	268/24	166/66	chr.rRNA5
chr	3140943	G/A	267/25	166/62	chr.rRNA5
chr	3141614	C/A	793/483	437/345	chr.rRNA5
chr	3141615	C/G	794/463	445/337	chr.rRNA5
chr	3141616	A/G	776/481	431/344	chr.rRNA5
chr	3141621	T/A	763/475	417/341	chr.rRNA5
chr	3141624	T/C	779/473	435/331	chr.rRNA5
chr	3141625	G/C	754/480	422/346	chr.rRNA5
chr	3141626	G/C	764/486	431/346	chr.rRNA5
chr	3141968	T/C	749/590	673/493	chr.rRNA5
chr	3141970	C/G	737/587	689/491	chr.rRNA5
chr	3141971	C/T	735/586	684/487	chr.rRNA5
chr	3141972	A/C	697/583	640/493	chr.rRNA5
chr	3141973	C/T	729/576	692/474	chr.rRNA5
chr	3141982	G/A	730/556	672/460	chr.rRNA5
chr	3141983	C/A	736/557	687/461	chr.rRNA5
chr	3141985	G/C	731/563	686/465	chr.rRNA5
chr	3141987	A/G	718/562	663/464	chr.rRNA5
chr	3141988	G/A	730/563	681/463	chr.rRNA5
chr	3142263	C/A	79/376	89/291	chr.rRNA5
chr	3142264	A/C	55/413	63/330	chr.rRNA5
chr	3142265	C/A	59/376	65/300	chr.rRNA5
chr	3142269	C/G	83/380	85/292	chr.rRNA5
chr	3142270	C/G	61/390	65/302	chr.rRNA5
chr	3142277	A/C	70/345	64/307	chr.rRNA5
chr	3142278	G/C	89/346	88/304	chr.rRNA5
chr	3142281	A/C	94/349	85/309	chr.rRNA5
chr	3142282	T/A	69/361	62/324	chr.rRNA5
chr	3142283	T/C	87/347	85/299	chr.rRNA5
chr	3142286	G/T	82/362	68/328	chr.rRNA5
chr	3142287	T/G	81/368	65/327	chr.rRNA5
chr	3142288	G/C	107/345	89/306	chr.rRNA5
chr	3142910	A/C	304/262	183/151	chr.rRNA5
chr	3142912	A/C	468/137	232/81	chr.rRNA5
chr	3142913	C/G	467/261	233/149	chr.rRNA5
chr	3142915	G/T	294/263	170/150	chr.rRNA5
chr	3142916	T/C	455/260	221/149	chr.rRNA5
chr	3142917	G/C	457/259	226/147	chr.rRNA5
chr	3142919	G/A	547/160	320/56	chr.rRNA5
chr	3142921	A/G	550/159	313/55	chr.rRNA5
chr	3142922	C/G	461/258	229/148	chr.rRNA5
chr	3142924	C/A	304/261	167/153	chr.rRNA5
chr	3142925	T/C	474/261	219/151	chr.rRNA5
chr	3142926	G/C	440/262	211/153	chr.rRNA5
chr	3142927	C/A	291/260	163/148	chr.rRNA5

chr	3142929	T/G	441/260	207/151	chr.rRNA5
chr	3143109	C/A	2168/703	2355/425	chr.rRNA5
chr	3143110	A/C	2160/703	2357/400	chr.rRNA5
chr	3143111	A/C	2158/700	2364/408	chr.rRNA5
chr	3143117	G/A	2067/688	2303/399	chr.rRNA5
chr	3143128	A/T	2133/593	2333/336	chr.rRNA5
chr	3143187	T/C	2625/1000	2587/470	chr.rRNA5
chr	3143188	C/A	2645/977	2592/466	chr.rRNA5
chr	3143190	A/C	2674/980	2619/464	chr.rRNA5
chr	3143191	C/A	2692/972	2622/453	chr.rRNA5
chr	3143194	G/A	2691/980	2644/469	chr.rRNA5
chr	3143195	G/A	2703/955	2634/455	chr.rRNA5
chr	3143201	A/T	2698/1070	2595/523	chr.rRNA5
chr	3143206	A/G	2662/1071	2537/547	chr.rRNA5
chr	3143207	C/T	2660/1060	2572/520	chr.rRNA5
chr	3143213	C/A	2619/1100	2539/537	chr.rRNA5
chr	3143224	G/C	2623/1149	2534/558	chr.rRNA5
chr	3143232	C/T	2631/1151	2539/546	chr.rRNA5
chr	3143234	C/A	2647/1169	2549/561	chr.rRNA5
chr	3143235	A/G	2634/1160	2537/560	chr.rRNA5
chr	3143236	G/C	2683/1168	2539/559	chr.rRNA5
chr	3144446	T/A	5/6	0/0	chr.rRNA6
chr	3144577	C/T	473/236	170/156	chr.rRNA6
chr	3145197	G/T	1927/930	907/484	gene2698,
chr	3145198	A/G	1913/932	907/491	gene2698,
chr	3145199	C/A	1939/943	911/494	gene2698,
chr	3145200	C/G	1900/937	913/491	gene2698,
chr	3145205	G/C	1898/916	920/487	gene2698,
chr	3145206	G/T	1910/918	930/487	gene2698,
chr	3145208	C/A	1908/917	931/488	gene2698,
chr	3145209	G/A	1927/918	933/487	gene2698,
chr	3145216	A/G	1956/970	952/519	gene2698,
chr	3151485	C/T	18/8	17/1	gene2704
chr	3152987	A/G	8/12	16/0	gene2706
chr	3234448	G/C	55/32	81/33	gene2777
chr	3291773	C/T	22/8	142/0	-
chr	3388274	A/C	87/33	111/20	-
chr	3388276	G/C	61/31	91/28	-
chr	3435360	T/G	11/6	42/0	-
chr	3585869	G/A	22/0	0/64	gene3072
chr	3586364	G/A	37/0	0/18	-
chr	3586365	G/A	37/0	0/18	-
chr	3611909	C/T	23/7	10/0	gene3097
chr	3696327	G/A	3/13	0/0	gene3180
chr	3766915	A/C	180/74	458/148	gene3240
chr	3877455	C/T	9/6	33/1	gene3341
chr	3918588	T/G	0/45	0/180	-
chr	3926574	A/C	21/11	58/19	-
chr	3993163	T/C	40/0	16/7	gene3444
chr	4026665	G/A	199/0	0/187	gene3469
chr	4026666	G/A	199/0	0/188	gene3469
chr	4037929	G/A	444/171	177/85	chr.rRNA7
chr	4040218	C/G	88/187	52/127	chr.rRNA8
chr	4040219	A/C	81/193	43/135	chr.rRNA8
chr	4040220	C/A	81/195	44/135	chr.rRNA8
chr	4040223	A/G	78/186	52/125	chr.rRNA8
chr	4040224	A/T	72/188	37/134	chr.rRNA8
chr	4040225	T/G	76/188	49/127	chr.rRNA8
chr	4040228	C/G	73/189	41/126	chr.rRNA8
chr	4040229	T/G	75/188	38/128	chr.rRNA8
chr	4040236	G/C	71/196	44/126	chr.rRNA8

chr	4040237	G/C	75/188	53/124	chr.rRNA8
chr	4040241	G/T	77/185	44/122	chr.rRNA8
chr	4040242	T/G	70/204	42/139	chr.rRNA8
chr	4040243	G/T	80/187	54/122	chr.rRNA8
chr	4040518	C/T	585/351	637/263	chr.rRNA8
chr	4040519	T/C	585/353	622/272	chr.rRNA8
chr	4040521	C/G	594/352	655/270	chr.rRNA8
chr	4040523	G/T	587/353	646/272	chr.rRNA8
chr	4040524	C/T	590/351	640/268	chr.rRNA8
chr	4040533	G/A	608/377	653/285	chr.rRNA8
chr	4040534	T/G	576/386	611/297	chr.rRNA8
chr	4040535	G/A	615/381	652/292	chr.rRNA8
chr	4040536	G/C	621/384	646/294	chr.rRNA8
chr	4040538	A/G	616/380	649/294	chr.rRNA8
chr	4040880	C/G	681/187	430/133	chr.rRNA8
chr	4040881	C/G	673/186	423/134	chr.rRNA8
chr	4040882	A/G	683/182	432/131	chr.rRNA8
chr	4040885	A/T	661/186	421/135	chr.rRNA8
chr	4040890	T/C	671/191	423/136	chr.rRNA8
chr	4040891	G/C	685/179	431/131	chr.rRNA8
chr	4040892	G/T	690/190	432/136	chr.rRNA8
chr	4077441	G/T	27/7	127/0	gene3512
chr	4077442	G/T	27/7	127/0	gene3512
chr	4155332	G/A	0/0	5/8	gene3597
chr	4198188	T/C	150/51	248/62	gene3636
chr	4264532	T/G	12/5	25/8	-
chr	4316970	G/T	5/9	0/0	-
chr	4365702	G/A	33/0	0/12	-
chr	4367572	G/A	204/0	2/980	gene3781
chr	4424351	T/A	0/0	0/10	gene3843
chr	4463941	C/G	29/9	181/14	gene3883
chr	4499161	G/A	25/7	64/0	gene3914
chr	4597016	T/G	43/19	249/45	gene3996
chr	4609695	A/C	80/23	219/41	gene4007
chr	4630002	G/A	458/194	185/79	chr.rRNA10
chr	4630910	C/T	5/6	11/4	-
chr	4632291	C/G	103/153	67/162	chr.rRNA11
chr	4632292	A/C	94/159	55/172	chr.rRNA11
chr	4632293	C/A	94/160	54/170	chr.rRNA11
chr	4632296	A/G	88/152	66/161	chr.rRNA11
chr	4632297	A/T	79/154	52/168	chr.rRNA11
chr	4632298	T/G	85/151	64/160	chr.rRNA11
chr	4632301	C/G	82/158	54/160	chr.rRNA11
chr	4632302	T/G	82/154	53/162	chr.rRNA11
chr	4632309	G/C	81/166	59/162	chr.rRNA11
chr	4632310	G/C	87/159	70/154	chr.rRNA11
chr	4632314	G/T	83/165	61/157	chr.rRNA11
chr	4632315	T/G	83/174	54/174	chr.rRNA11
chr	4632316	G/T	88/154	71/153	chr.rRNA11
chr	4632591	C/T	609/329	554/272	chr.rRNA11
chr	4632592	T/C	599/334	546/273	chr.rRNA11
chr	4632594	C/G	614/334	562/272	chr.rRNA11
chr	4632596	G/T	606/332	554/271	chr.rRNA11
chr	4632597	C/T	604/328	549/273	chr.rRNA11
chr	4632606	G/A	625/347	561/285	chr.rRNA11
chr	4632607	T/G	587/359	519/293	chr.rRNA11
chr	4632608	G/A	631/358	558/290	chr.rRNA11
chr	4632609	G/C	633/351	565/290	chr.rRNA11
chr	4632611	A/G	637/358	553/293	chr.rRNA11
chr	4632953	C/G	713/216	417/141	chr.rRNA11
chr	4632954	C/G	697/216	417/141	chr.rRNA11

chr	4632955	A/G	718/211	420/142	chr.rRNA11
chr	4632958	A/T	699/208	401/139	chr.rRNA11
chr	4632963	T/C	708/218	410/145	chr.rRNA11
chr	4632964	G/C	720/216	423/140	chr.rRNA11
chr	4632965	G/T	715/221	423/143	chr.rRNA11
chr	4685740	C/T	1/9	0/0	gene4058
chr	4718308	T/C	2337/2250	2296/1800	gene4083
chr	4718311	C/T	2954/2052	2854/1600	gene4083
chr	4718389	C/T	4379/1165	4591/991	gene4083
chr	4718392	T/C	4073/1505	4351/1207	gene4083
chr	4773216	A/C	36/9	142/16	-
chr	4773223	T/G	46/16	165/31	-
chr	4777392	T/G	0/0	25/11	gene4134
chr	4797383	G/C	47/56	83/38	gene4151
chr	4809595	G/A	142/0	0/359	gene4163
chr	4817791	A/C	3/5	8/6	-
chr	4856109	A/C	16/7	27/2	-
chr	4874998	C/T	0/0	8/6	gene4225
chr	4951074	G/A	16/0	3/7	-
chr	5119723	G/C	26/9	34/10	-
chr	5230940	G/A	5/6	9/0	-
chr	5341143	C/T	11/6	70/0	gene4616
chr	5368059	G/T	0/9	0/0	gene4641
chr	5380085	G/A	6/6	0/0	gene4646
chr	5413285	T/G	225/105	89/68	gene4682
chr	5414020	T/G	56/14	39/7	gene4683
chr	5454681	G/A	0/0	13/7	gene4721
chr	5497368	C/G	99/0	0/69	gene4758
chr	5519590	A/G	21/7	20/0	gene4775
chr	5580285	T/G	13/1	44/15	-
chr	5580288	G/T	5/6	53/0	-
chr	5580303	A/C	0/0	9/21	-
chr	5580304	G/C	0/0	6/18	-
chr	5591494	C/G	50/42	93/34	gene4833
chr	5627290	C/T	52/0	0/63	gene4865
chr	5650378	C/G	62/76	38/19	gene4886
chr	5664236	G/A	15/7	40/0	gene4898
chr	5677642	C/T	1487/4	0/1982	gene4910
chr	5678549	A/G	10/6	64/0	gene4911
chr	5680533	C/T	8/0	8/6	gene4912
chr	5723193	A/C	4/6	7/5	-
chr	5723203	T/G	3/7	10/7	-
chr	5782737	C/G	0/0	8/6	gene5003
chr	5786678	T/C	29/0	0/36	gene5008
chr	5832140	A/G	26/8	46/0	gene5042
chr	6001961	C/T	796/0	0/1768	gene5194
chr	6024141	T/G	16/3	28/9	gene5217
chr	6025389	A/C	12/6	34/16	gene5218
chr	6049380	C/A	2/6	14/0	-
chr	6098489	G/A	440/209	220/91	chr.rRNA13
chr	6099434	A/G	46/23	104/68	-
chr	6099435	G/C	47/24	107/69	-
chr	6099438	T/G	45/25	93/66	-
chr	6099441	G/T	49/22	101/52	-
chr	6100776	C/G	95/184	67/154	chr.rRNA14
chr	6100777	A/C	86/186	51/170	chr.rRNA14
chr	6100778	C/A	87/187	52/169	chr.rRNA14
chr	6100781	A/G	81/183	57/156	chr.rRNA14
chr	6100782	A/T	76/184	43/163	chr.rRNA14
chr	6100783	T/G	81/185	57/156	chr.rRNA14
chr	6100786	C/G	79/188	45/156	chr.rRNA14

chr	6100787	T/G	77/188	43/157	chr.rRNA14
chr	6100794	G/C	76/199	47/157	chr.rRNA14
chr	6100795	G/C	84/191	59/150	chr.rRNA14
chr	6100799	G/T	77/187	45/153	chr.rRNA14
chr	6100800	T/G	71/210	42/173	chr.rRNA14
chr	6100801	G/T	85/189	62/151	chr.rRNA14
chr	6101076	C/T	603/333	580/261	chr.rRNA14
chr	6101077	T/C	599/335	567/260	chr.rRNA14
chr	6101079	C/G	605/336	584/260	chr.rRNA14
chr	6101081	G/T	597/333	579/258	chr.rRNA14
chr	6101082	C/T	601/333	572/259	chr.rRNA14
chr	6101091	G/A	611/353	584/282	chr.rRNA14
chr	6101092	T/G	592/360	539/284	chr.rRNA14
chr	6101093	G/A	618/356	577/281	chr.rRNA14
chr	6101094	G/C	618/356	582/284	chr.rRNA14
chr	6101096	A/G	627/358	563/284	chr.rRNA14
chr	6101438	C/G	716/186	424/138	chr.rRNA14
chr	6101439	C/G	710/185	418/140	chr.rRNA14
chr	6101440	A/G	722/184	429/135	chr.rRNA14
chr	6101443	A/T	692/184	416/132	chr.rRNA14
chr	6101448	T/C	709/194	427/140	chr.rRNA14
chr	6101449	G/C	724/189	433/135	chr.rRNA14
chr	6101450	G/T	723/193	433/138	chr.rRNA14
chr	6205463	T/C	4/8	14/0	gene5377
chr	6316982	C/G	1372/456	1694/517	-
chr	6321856	A/T	1885/2097	875/2678	gene5493
chr	6321864	A/T	1914/184	892/289	gene5493
chr	6334458	C/G	4/6	37/3	gene5505
chr	6355194	C/T	2/6	11/0	gene5522
chr	6409207	G/A	8/6	120/0	gene5572
chr	6470733	C/G	12/1	65/22	-
chr	6507678	T/C	12/0	22/7	gene5661
chr	6570832	T/G	4/7	24/0	gene5719
chr	6597667	T/C	2/12	12/0	-
chr	6658299	G/A	425/189	195/94	chr.rRNA16
chr	6660588	C/G	77/190	63/164	chr.rRNA17
chr	6660589	A/C	71/196	51/175	chr.rRNA17
chr	6660590	C/A	71/196	51/174	chr.rRNA17
chr	6660593	A/G	71/189	48/163	chr.rRNA17
chr	6660594	A/T	62/193	38/172	chr.rRNA17
chr	6660595	T/G	69/190	48/164	chr.rRNA17
chr	6660598	C/G	64/198	40/170	chr.rRNA17
chr	6660599	T/G	66/199	41/168	chr.rRNA17
chr	6660606	G/C	63/213	44/164	chr.rRNA17
chr	6660607	G/C	70/204	62/158	chr.rRNA17
chr	6660611	G/T	68/206	43/161	chr.rRNA17
chr	6660612	T/G	57/218	39/183	chr.rRNA17
chr	6660613	G/T	75/205	63/162	chr.rRNA17
chr	6660888	C/T	592/338	624/274	chr.rRNA17
chr	6660889	T/C	585/337	608/278	chr.rRNA17
chr	6660891	C/G	593/335	625/274	chr.rRNA17
chr	6660893	G/T	586/335	615/274	chr.rRNA17
chr	6660894	C/T	586/332	617/271	chr.rRNA17
chr	6660903	G/A	601/355	629/296	chr.rRNA17
chr	6660904	T/G	571/360	589/299	chr.rRNA17
chr	6660905	G/A	600/354	627/297	chr.rRNA17
chr	6660906	G/C	606/357	635/299	chr.rRNA17
chr	6660908	A/G	608/360	633/302	chr.rRNA17
chr	6661250	C/G	720/202	457/151	chr.rRNA17
chr	6661251	C/G	700/200	455/150	chr.rRNA17
chr	6661252	A/G	719/196	461/152	chr.rRNA17

chr	6661255	A/T	704/196	438/147	chr.rRNA17
chr	6661260	T/C	705/200	454/156	chr.rRNA17
chr	6661261	G/C	723/194	462/151	chr.rRNA17
chr	6661262	G/T	721/202	464/155	chr.rRNA17
chr	6703432	T/G	44/15	22/4	gene5815
chr	6703440	A/C	15/37	10/26	gene5815
chr	6703449	A/C	9/31	7/15	gene5815
chr	6850700	C/T	340/0	0/888	gene5981
chr	6913609	T/G	26/11	97/26	gene6040
chr	6952724	G/C	12/6	49/5	gene6074
chr	7111474	A/G	30/8	24/0	gene6226
chr	7152884	A/C	0/0	20/7	-
chr	7152891	A/C	0/0	17/16	-
chr	7167600	T/C	2/6	0/0	gene6285
chr	7260769	T/C	7/6	9/0	gene6374
chr	7273869	G/T	16/8	43/0	gene6385
chr	7275049	G/A	6/6	33/0	gene6386
chr	7318360	T/G	0/16	1/12	-
chr	7318366	T/G	0/10	0/0	-
chr	7318372	A/C	0/14	2/10	-
chr	7377272	G/C	10/6	13/0	gene6479
chr	7377693	C/T	18/13	45/0	gene6479
chr	7423697	T/A	34/12	128/23	gene6518
chr	7472117	T/G	0/0	24/13	-
chr	7472123	T/G	0/0	23/18	-
chr	7472130	T/C	14/4	89/24	-
chr	7472134	G/C	12/4	67/45	-
chr	7559606	T/C	21/9	41/0	gene6636
chr	7575500	G/A	25/10	26/0	gene6652
chr	7658301	T/G	18/3	36/10	-
chr	7676237	C/G	7/9	11/0	-
chr	7693839	T/A	0/0	25/7	-
chr	7799612	G/C	0/11	0/0	gene6868
plasmid	26627	T/G	41/0	0/51	plasmid_32

Table S6. Raw results of InDels analysis for strains 891 and 891-B6.

CHROM	POS	REF/ALT	891	891-B6	GENE_ID
chr	204256	C/CG	44795	0/0	gene197
chr	1011326	G/GCACGAATAAAA	44749	0/0	gene905
chr	1876077	C/CG	22/20	14062	-
chr	1878545	CA/C	48/0	44903	gene1578
chr	1948422	AG/A	535/192	356/90	chr.rRNA2
chr	1948431	AC/A	569/175	375/84	chr.rRNA2
chr	1948638	A/ACG	2083/671	2414/480	chr.rRNA2
chr	1949785	AG/A	487/194	212/70	chr.rRNA3
chr	2224772	GCA/G	3/6	0/0	gene1850
chr	2310314	G/GCA	2/7	10/0	gene1924
chr	2972609	GC/G	16/40	5/27	-
chr	3067116	G/GAAATGAACTCA	28/7	19/0	-
chr	3142914	AG/A	553/166	321/57	chr.rRNA5
chr	3142923	AC/A	587/159	332/53	chr.rRNA5
chr	3143130	A/ACG	2102/716	2315/431	chr.rRNA5
chr	3144277	AG/A	473/197	222/67	chr.rRNA6
chr	3489411	C/CGTG	11/11	139/23	gene2977
chr	3663103	AG/A	35/0	32/51	-
chr	4086595	A/AC	0/0	9/6	-
chr	4160463	A/ATTC	14/11	40/0	gene3602
chr	4476383	CG/C	4/7	0/0	gene3893
chr	4487150	G/GC	8/9	10/0	-
chr	4718277	A/ACGC	2541/1068	2543/882	gene4083
chr	4724798	C/CG	24/5	17/68	-
chr	5578007	GCA/G	55/9	117/49	gene4820
chr	6146087	G/GAGC	10/0	4/6	-
chr	6209271	C/CA	6/6	33/0	gene5386
chr	6658601	CG/C	479/122	216/33	chr.rRNA16
chr	6880582	TC/T	50/206	121/9	-
chr	6981413	T/TA	13/6	16/0	gene6103
chr	7377277	G/GC	10/6	13/0	gene6479
chr	7432635	AC/A	20/10	28/0	gene6529
chr	7438526	G/GC	10/6	0/0	gene6538
